# NR4A1 suppresses breast cancer growth by repressing c-Fos-mediated lipid and redox dyshomeostasis

**DOI:** 10.1038/s12276-025-01430-3

**Published:** 2025-04-01

**Authors:** Cen Jiang, Youzhi Zhu, Junsi Zhang, Huaying Chen, Weiwei Li, Ruiwang Xie, Lingjun Kong, Ling Chen, Xiangjin Chen, Huifang Huang, Sunwang Xu

**Affiliations:** 1 Central Laboratory, Fujian Medical University Union Hospital, Fuzhou, China; 2 Department of Thyroid and Breast Surgery, the First Affiliated Hospital, Fujian Medical University, Fuzhou, China; 3 Department of Thyroid and Breast Surgery, National Regional Medical Center, Binhai Campus of the First Affiliated Hospital, Fujian Medical University, Fuzhou, China; 4 Fujian Provincial Key Laboratory of Precision Medicine for Cancer, Fuzhou, China

**Keywords:** Breast cancer, Cell growth, Epigenetics

## Abstract

The specific function of NR4A1 as a transcriptional regulator in cancer remains unclear. Here we report the biological effect of NR4A1 in suppressing breast cancer (BC) growth. We found that NR4A1 deficiency was correlated with BC progression in the clinic. Genetic deletion of NR4A1 in BC cells significantly promoted cellular proliferation and tumor growth. Moreover, global metabolome screening indicated that the deletion of NR4A1 resulted in tumor lipid remodeling and phospholipid accumulation, which was accompanied by increases in fatty acid and lipid uptake. In addition, NR4A1 knockout induced oxidative stress that aggravated redox balance disruption. Mechanistically, transcriptomic and epigenomic analyses revealed that NR4A1 restrained BC cell proliferation by directly interacting with c-Fos and competitively inhibiting c-Fos binding to the promoter of the target gene PRDX6, which is involved in lipid and redox homeostasis. Notably, we confirmed that the treatment of BC cells with the selective NR4A1 agonist cytosporone B significantly activated the expression of NR4A1, followed by increased interaction between NR4A1 and c-Fos, thereby interfering with c-Fos-mediated transcriptional regulation of BC cell growth. Thus, NR4A1 plays a vital role in reducing the c-Fos-induced activation of downstream signaling cascades in BC, suggesting that agents that activate NR4A1 may be potential therapeutic strategies.

## NR4A1 deficiency fuels breast cancer growth and progression

NR4A1 is a protein that plays a role in various cell processes, including growth and survival. Researchers explored how NR4A1 affects breast cancer (BC) growth and metabolism. They used laboratory-grown cells and animal models to study this. They found that NR4A1 can suppress tumor growth by interacting with another protein called c-Fos, which is involved in gene regulation. This interaction affects the activity of a gene called PRDX6, which is linked to lipid metabolism and redox balance. The study showed that, when NR4A1 is absent, BC cells grow more due to changes in lipid metabolism and increased oxidative stress. Researchers concluded that activating NR4A1 could be a potential strategy for BC treatment. This work highlights the importance of understanding NR4A1’s role in cancer and suggests that targeting it could help to manage BC progression in the future.

This summary was initially drafted using artificial intelligence, then revised and fact-checked by the author.

## Introduction

As an orphan nuclear receptor, NR4A1 (Nur77, TR3 or NGFIB) belongs to the steroid/thyroid/retinoid receptor superfamily^1,2^. When various intracellular and extracellular stimuli are present, NR4A1 plays a critical role in multiple biological processes, such as cell growth, DNA repair, metabolism and survival^3–7^. In addition to its nongenomic activities, such as regulating cell mitophagy and apoptosis^8,9^, the transcriptional regulation of target genes by NR4A1 has been highlighted in several studies. For example, the NR4A1-dependent transcriptional program broadly regulates vascular permeability in response to progesterone in the vascular endothelium^10^. Furthermore, genome-wide epigenetic and gene expression studies in tolerant T cells revealed that NR4A1 acts as a key mediator of T cell dysfunction^11^. As a transcription factor, NR4A1 can directly bind to a specific DNA responsive element, named NGFI-B response element (NBRE), alone or with its heterodimeric partner^12^, thus activating or inhibiting the transcription of specific genes.

NR4A1 is differentially expressed in multiple cancer types and has cancer type-specific biological functions that are dependent on its transcriptional targets^13,14^. For example, NR4A1 functions as a tumor suppressor, and its abrogation in mice led to mixed myelodysplastic/myeloproliferative neoplasms^15^. Nevertheless, NR4A1 knockdown in pancreatic cancer cells decreased proliferation and induced apoptosis by decreasing the expression of antiapoptotic genes^16^. Previously, we showed that NR4A1 promotes the pathogenesis of papillary thyroid cancer by increasing the transcriptional activation of the oncogene LEF1 (ref. ^17^). Thus, NR4A1 has pleiotropic regulatory effects on cancers, which are dependent on the expression of specific genes that are under the transcriptional control of NR4A1 in different contexts.

Breast cancer (BC) has become a major threat to women’s health worldwide^18,19^. Despite enormous improvements in treatment, there is still an urgent need to identify the vital genes and mechanisms responsible for tumor progression on a genome-wide scale^20–22^. The role of NR4A1 in BC remains controversial. Inflammation-induced NR4A1 is a strong activator of TGF-β signaling that promotes BC cell migration and invasion^23^; furthermore, NR4A1 promotes BC cell tumorigenesis by transcriptionally regulating immediate early genes under replication stress^4^. However, ectopic expression of NR4A1 in a triple-negative BC cell line with low endogenous NR4A1 inhibited cell growth^24^. Alternatively, the presence of NR4A1 in mammary tissues inhibited mammary tumor development^25^. Given the conflicting evidence, it is critical to clarify the molecular mechanisms underlying the different biological functions of NR4A1 in BC.

As a hallmark of cancer progression, metabolic reprogramming is complex and exhibits cell- and tissue type-specific characteristics^26^. NR4A1 has multifaceted regulatory effects on glucose and lipid metabolism. NR4A1 can lead to ATP depletion and cell growth arrest in hepatocellular carcinoma by interacting with the rate-limiting enzyme to increase gluconeogenesis and suppress glycolysis^27^. In addition, NR4A1 facilitated metabolic adaptation in melanoma cells upon glucose deprivation to support tumor cell survival^28^. Metabolic disorders often occur alongside BC^29–31^. Metastatic triple-negative BC maintains high levels of ATP through fatty acid β-oxidation^32^. Specific silencing of either acetyl-CoA carboxylase alpha or the fatty acid synthase genes in BC results in the induction of apoptosis^33^. Nonetheless, whether and how NR4A1 preprograms metabolic processes in BC cells remains unclear.

In this study, we investigated the relationships between BC growth and alterations in lipid metabolism and redox balance based on NR4A1 regulation. We found that NR4A1 suppressed tumor growth in BC by antagonizing the c-Fos-mediated transcriptional activation of PRDX6, a bifunctional enzyme involved in cellular lipid and redox homeostasis. Importantly, we confirmed that pharmacologic activation of NR4A1 could be an efficient strategy for BC therapy. In summary, this work provides evidence of the involvement of NR4A1–c-Fos–PRDX6 signaling in hindering BC progression.

## Materials and methods

### Cell lines

MCF7, HEK293T, SKBR3 and MDA-MB-231 cells were cultured in DMEM medium (Hyclone, cat. no. SH30243.01). T47D cells were cultured in RPMI-1640 medium (Hyclone, cat. no. SH30809.01). MCF10A cells were cultured in DMEM/F12 medium (Hyclone, cat. no. SH30023.01) supplemented with 5% horse serum, 20 ng/ml epidermal growth factor, 10 mg/ml insulin and 0.5 mg/ml hydrocortisone. The DMEM and RPMI-1640 media were supplemented with 10% fetal bovine serum (Gibco, cat. no. 10270-106), 100 U/ml penicillin and 100 mg/ml streptomycin. All cell lines were obtained from the American Type Culture Collection, cultured at 37 °C in a humidified atmosphere with 5% CO_2_ and ensured to be mycoplasma-negative cultures by monthly mycoplasma tests.

### sgRNA-mediated knockout cell generation

For NR4A1 stable knockout using CRISPR single guide RNAs (sgRNAs), sgRNAs were first cloned into LentiCRISPR-V2 vector (Addgene, cat. no. 52961) using BsmBI sites. The constructs were than packaged using the lentiviral packaging system to transfect the constructs with packaging plasmids (psPAX and pMD2.G) into 293T cells. Lentivirus was collected 48 h after transfection and passed through a 0.45-mm filter. The lentivirus-infected cells were selected by treatment with 2 mg/ml puromycin until puromycin-resistant stable cells were generated. The sgRNA sequences for NR4A1 knockout were as follows: NR4A1-sg1, TACACCCGTGACCTCAACCA; NR4A1-sg2, GGCTAACAAGGACTGCCCTG. The NR4A1 knockout efficiency was validated by immunoblotting.

For the deletion of NBRE-motif-containing region in the PRDX6 promoter, two independent sgRNAs (targeting the second NBRE motif by NBRE-sg1 (CCGTACCAATGTCAGTGCTA) and the third NBRE motif by NBRE-sg2 (GTGGCAAGACCCAGAAGTAT), respectively) were cloned into a modified lentiCRISPR-V2 vector, which carries two independent sgRNAs. The lentivirus containing this modified dual sgRNAs construct was packed and infected to MCF7 cells. The puromycin-resistant single-cell clones were isolated and validated via PCR followed by Sanger sequencing. Primers for NBRE motif deletion validation PCR were F 5′-AAAGTTTAGTTACATTACCC-3′ and R 5′-GCTACGATGAACTGTCTAAG-3′.

### Cell proliferation assay

A total of 5000 cells were seeded in 96-well plates containing 100 μl regular full-serum medium. Ten microlitres of Cell Counting Kit-8 (CCK-8) solution (Dojindo, cat. no. CK04) was added to the cells at the indicated time points and incubated at 37 °C for 1 h. For Csn-B (Selleck, cat. no. S6674) treatment assays, the cells were treated with indicated drug concentrations or vehicle control. The proliferation curves were determined by calculating the relative value of absorbance measured at 450 nm on a microplate reader.

### Colony formation assay

A total of 500 cells in 2 ml of complete medium were seeded into six-well plates, which were kept at 37 °C in an incubator with 5% CO_2_ until the colonies were visible. Colonies were fixed with methanol for 15 min and stained with 0.1% of crystal violet for 10 min. ImageJ software was used to count the colony number.

### Cell migration and invasion assay

MCF10A cells were starved without fetal bovine serum for 4 h, then resuspended with a concentration of 20,000 cells in 100 μl culture medium without serum and cultured in the upper chamber of noncoated transwell inserts (for migration assay) or Matrigel gel precoated transwell insert (for invasion assay) in the 12-well plate. A total of 500 μl culture medium with serum was added in the lower chamber and used as a chemoattractant to encourage cell migration or invasion. After 24 h incubation at 37 °C, all cells were stained with 0.1% crystal violet and the nonmigrated or noninvaded cells were gently removed by cotton swab. The migrated and invaded cells were photographed and counted under an inverted microscope in five fields.

### EdU incorporation assay

Cells were seeded into six-well plates and incubated overnight. The 5-ethynyl-2′-deoxyuridine (EdU) incorporation assay kit (Beyotime, cat. no. C0071S) was used to evaluate cell proliferation. The cells were examined on the BD FACSCelesta Cell Analyzer (BD Biosciences).

### Lipid uptake assay

Cells were seeded in six-well plates and incubated overnight. The next day, cells were washed with cold phosphate-buffered saline (PBS) and 2 μM 4,4-Difluoro-5,7-Dimethyl-4-Bora-3a,4a-Diaza-s-Indacene-3-Hexadecanoic Acid (BODIPY FL C16) staining solution (Thermo Fisher Scientific, cat. no. D3821) were added. Cells were then incubated in the dark for 15 min at 37 °C. The analyze was performed by the flow cytometry on the BD FACSCelesta Cell Analyzer (BD Biosciences).

### Measurement of ATP concentration

Cells were seeded in a six-well plate and maintained in medium overnight. These cells were then lysed in 200 μl lysis buffer from the ATP Assay Kit (Beyotime, cat. no. S0026). The absorbance was measured by a spectrophotometer plate reader according to the manufacturer’s instructions. All values were normalized to the amount of protein.

### Metabolic flux analysis

The real-time oxygen consumption rate (OCR) rates and extracellular acidification rate (ECAR) were determined using the Seahorse Extracellular Flux (XFe24) analyzer (Agilent) with the Seahorse XF Cell Mito Stress Test Kit (Agilent, cat. no. 103015-100) and Seahorse XF Glycolysis Stress Test Kit (Agilent, cat. no. 103020-100) according to the manufacturer’s user guide. In brief, cells were seeded into cell culture plates and incubated overnight to allow cell attachment at the same density (80–90% confluence). Then, the medium was changed to prewarmed Seahorse XF assay medium (pH 7.4) supplemented with 2 mM glutamine, 10 mM glucose and 1 mM pyruvate (for OCR measurement) or supplemented with 2 mM glutamine (for ECAR measurement) and incubated in non-CO_2_ incubator at 37 °C for 1 h before the assay. Injections of oligomycin (1 μM), FCCP (0.5 μM) and rotenone/antimycin A (0.5 μM) were loaded into injection ports, respectively, for OCR. Injections of glucose (10 mM), oligomycin (1 μM) and 2-Deoxy-D-glucose (2-DG) (50 mM) were loaded into injection ports, respectively, for ECAR. Each sample was assayed on the basis of three replicates. The results were normalized to the protein content each well.

### ROS and glutathione measurement

The production of reactive oxygen species (ROS) was measured using the Dichlorodihydrofluorescein diacetate (DCFH-DA) (Beyotime, cat. no. S0033M) method. In brief, cells were incubated with DCFH-DA (10 μM) for 20 min at 37 °C. After the extracellular dye was removed, the cells were washed three times. Subsequently, the cells were analyzed by the flow cytometry. Glutathione (GSH) and Glutathione disulfide (GSSG) levels were measured using a GSH and GSSG Assay Kit (Beyotime, cat. no. S0053) according to the manufacturer’s instructions.

### Lipid peroxidation assay

The level of lipid peroxidation was assessed using the Lipid Peroxidation Assay Kit (Abcam, cat. no. ab243377). In brief, cells were stained with 1× Lipid Peroxidation Reagent for 30 min in complete growth medium at 37 °C, followed by washing and fluorescence detection by flow cytometry. As the sensitive Lipid Peroxidation Sensor changes its fluorescence from red to green upon peroxidation by ROS in cells, the data were represented as the ratios of red (phosphatidylethanolamine (PE))/green (FITC) fluorescence intensities.

### Immunoblotting

Total cell lysates were resuspended in lysis buffer (50 mM Tris-Cl pH 7.4, 1% Triton X-100, 1% sodium deoxycholate, 150 mM NaCl and 0.1% SDS) supplemented with 1% protease inhibitor cocktails (Sigma, cat. no. P8340), 1 mM phenylmethylsulfonyl fluoride (Sigma, cat. no. P7626). Protein concentrations were determined with a BCA protein assay (Beyotime, cat. no. P0012S) according to the instructions. Western blotting was performed for 10–20 μg protein using SDS–PAGE followed by transfer to polyvinylidene difluoride membranes (Millipore, cat. no. ZY101123). Primary antibody was incubated at 4 °C overnight. Secondary antibody was incubated for 1 h at room temperature. Membranes were visualized using the Bio-Rad ChemiDoc MP imaging system. Samples were used for immunoblotting analysis with antibodies against NR4A1 (Abcam, cat. no. 283264; 1:1,000 dilution), β-actin (Proteintech, cat. no. 66009-1-Ig; 1:5,000 dilution), CD36 (Abcam, cat. no. 133625; 1:1,000 dilution), hemagglutinin (HA) taq (Abcam, cat. no. 236632; 1:5,000 dilution), Flag (Abcam, cat. no. 205606; 1:5,000 dilution), PRDX6 (Abcam, cat. no. 133348; 1:2000 dilution) and c-Fos (Abcam, cat. no. 222699; 1:1,000 dilution).

### Co-IP assay

Cells were lysed with immunoprecipitation (IP) lysis buffer (20 mM Tris-Cl pH7.4, 1% Triton X-100, 1% sodium deoxycholate, 150 mM NaCl, 1 mM EDTA, 1 mM Na_3_VO_4_, 1% protease inhibitor cocktails and 1 mM phenylmethylsulfonyl fluoride). Then, the samples were centrifuged for 10 min at 12,000*g*, 4 °C, and the supernatant was recovered as the protein extract. Protein concentrations were determined with BCA protein assay according to the instructions. A total of 500 μg cell lysate was incubated with 2 μg indicated antibodies under gentle rotation at 4 °C overnight. Next, 20 μl of protein A-agarose beads (Santa Cruz Biotechnology, cat. no. sc-2001) was added and left with gentle rotation at 4 °C for 4 h. The beads were then washed five times in IP lysis buffer, and the bound proteins were evaluated by western blotting as described above. For the detection of immunoprecipitated protein without interference from denatured IgG, VeriBlot for IP Detection Reagent Horseradish Peroxidase (HRP) (Abcam, cat. no. ab131366; 1:4,000 dilution) was used.

### Luciferase assay

The wild-type or mutant PRDX6 promoter sequences and wild-type or mutant NR4A1 promoter sequences were amplified and cloned into pGL3-basic vector separately. Then, HEK293T cells were plated on a 12-well plate and co-transfected with wild-type or mutant luciferase plasmids and pRL-TK, and pcDNA3.0-c-Fos, pcDNA3.0-NR4A1 or pcDNA3.0-empty vector, for 24 h. The Dual Luciferase Reporter Gene Assay Kit (Yeasen, cat. no. 11402ES80) was used to measure the luciferase activity. The promoter activity was calculated by the ratio of Firefly luciferase to Renilla luciferase.

### Ultra-high performance liquid chromatography-quadrupole time-of-flight mass spectrometry (UHPLC–Q-TOF–MS) analysis

Cells were seeded into cell culture plates and incubated overnight to collect at a density of ~80–90% (six biological replicates). Extracts were flash frozen in liquid nitrogen. Untargeted metabolomics of samples was performed on an Agilent 1290 Infinity liquid chromatography system (Agilent Technologies) combined with a 6550 quadrupole time-of-flight mass spectrometer (Agilent Technologies). In brief, an ACQUITY HSS T3 UPLC column (2.1 mm × 100 mm, 1.8 μm, Waters) was maintained at 40 °C with a flow rate of 0.4 ml/min. Mobile phases were water (A) and acetonitrile (B), both containing 0.1% formic acid. The elution gradient started at 5% mobile phase B at 0–1 min increasing linearly to 90% B by 11 min, held at 90% B until 12 min, returning to 95% A for 2 min to recondition the column. The injection volume was 2 μl. The quadrupole time-of-flight mass spectrometer equipped with a dual jet stream electrospray ionization source was operated in negative and positive polarities. In positive ion mode (ESI+), the capillary voltage was set to 2.5 kV. In negative ion mode (ESI−), the capillary voltage was set to 1.5 kV. In both modes, the following settings were used: gas flow 8 l/min, fragmentor 135 V, gas temperature 325 °C, sheath temperature 325 °C, sheath flow 11 l/min and nebulizer 40 V. The metabolomics raw data were converted into mzML format using ProteoWizard software. Peak extraction, peak alignment and retention time correction were performed using the XCMS program. Metabolic identification information was obtained by searching the PubChem database, Kyoto Encyclopedia of Genes and Genomes (KEGG) database and the Human Metabolome database.

### RNA-seq and data analysis

RNA was extracted from NR4A1-knockout and empty-vector-infected parental control MCF7 cells using TRIzol reagent (Invitrogen, cat. no. 15596026) according to the manufacturer’s protocol. RNA sequencing (RNA-seq) library preparation used the Illumina TruSeq Stranded Total RNA Library Prep kit, and the sequencing was done on the Illumina HiSeq X Ten platform using paired-end 150-bp reads. Raw FASTQ reads were mapped to hg38 human genome using HISAT2. Quantification and differential analysis were performed using DESeq2 with default parameters. For gene set enrichment analysis (GSEA) of RNA-seq data, gene sets were downloaded from Molecular Signatures Database v2022.1, and GSEA was implemented using the GSEA software, with default parameters. The transcription factor enrichment analysis (TFEA) of different expressed genes in RNA-seq data was performed by using ChEA3 (https://maayanlab.cloud/chea3/).

### RNA extraction and RT-qPCR

Total RNA was extracted using TRIzol reagent (Invitrogen, cat. no. 15596026), and cDNA was obtained from the total RNA using cDNA Synthesis Kit (Yeasen, cat. no. 11141ES60). Quantitative real-time PCR (RT-qPCR) was conducted with qPCR SYBR Green Master Mix (Yeasen, cat. no. 11201ES08) on the ABI 7500 Real-Time PCR system. ACTB was used for normalization. The comparative threshold cycle (2^−ΔΔCT^) method was used to enable quantification of the mRNA of these genes. The primer sequences were as follows: PRDX6, F 5′-CGTGTGGTGTTTGTTTTTGG-3′, R 5′-TGCTGTCAGCTGGAGAGAGA-3′; CD36, F 5′-TGTGCAAAATCCACAGGAAG-3′, R 5′-CAGCGTCCTGGGTTACATTT-3′; MMP1, F 5′-AGGTCTCTGAGGGTCAAGCA-3′, R 5′-CTGGTTGAAAAGCATGAGCA-3′; LIF, F 5′-ACCAGATCAGGAGCCAACTG-3′, R 5′-GCCACATAGCTTGTCCAGGT-3′; CAMK2A, F 5′-AGCTGTCAGCCAGAGACCAT-3′, R 5′-TCCCTCCTCTGAGATGCTGT-3′; LEPR, F 5′-CCACCATTGGTACCATTTCC-3′, R 5′-CCTCATACGAAGACCCAGGA-3′; ACTB, F 5′-CATGTACGTTGCTATCCAGGC-3′, R 5′-CTCCTTAATGTCACGCACGAT-3′.

### ChIP–seq

To test c-Fos chromatin occupancy, chromatin immunoprecipitation followed by sequencing (ChIP–seq) for c-Fos was carried out. In brief, NR4A1-knockout and parental MCF7 cells were crosslinked with 1% formaldehyde in culture medium at room temperature for 10 min and quenched with 125 mM glycine. Then, the crosslinked cells were collected, lysed and sonicated. The cleared extract was subjected to IP with 3 μg of anti-c-Fos antibody (CST, cat. no. 2250) overnight at 4 °C with constant rotation. The isolated complexes were precipitated with protein A/G agarose beads. After washing, elution and reverse crosslinking, the ChIP DNA was purified and used to prepare ChIP–seq libraries by using NEBNext End Repair/dA-Tailing Module (NEB, cat. no. E7442) followed by adaptor ligation with NEBNext Ultra Ligation Module (NEB, cat. no. 4775). The ChIP and input DNA libraries were amplified for 15 cycles and sequenced using the Illumina NovaSeq 6000 platform with paired-end 1×75 as the sequencing mode. Raw FASTQ sequencing reads were mapped to hg38 human genome using Bowtie2 and uniquely mapped reads were processed further for peak identification. Peak calling over input control was performed using MACS 2.0 with a false discovery rate (FDR) value cutoff of 0.05 and normalization by signal per million reads. Differential ChIP peak analysis between groups was performed with DESeq2. Read pileups at genomic loci were imaged using Integrative Genomic Viewer. Genomic annotation of ChIP peaks was performed using ChIPseeker R package. Gene Ontology (GO) and KEGG enrichment of target genes was analyzed and visualized using ClusterProfiler.

### ChIP–qPCR

The ChIP DNA used for ChIP–qPCR was prepared by following the protocol of ChIP–seq, and the purified ChIP DNA was subjected to quantitative PCR analysis. The antibodies used for ChIP–qPCR were as follows: anti-c-Fos (CST, cat. no. 2250), anti-NR4A1 (Proteintech, cat. no. 12235-1-AP), anti-5mC (Sigma-Aldrich, cat. no. SAB2702243) and nonspecific IgG isotype (CST, cat. no. 3900). The primer sequences for ChIP–qPCR were as follows: PRDX6, F 5′-AATTGTGTTGAATATTTTATGGTTCA-3′, R 5′-AGCCCAGCTACGATGAACTG-3′; NR4A1, F 5′-TTGTATGGCCAAAGCTCGAC-3′, R 5′-CACTCCCCCAAGTTTCGTAG-3′.

### Bisulfite sequencing PCR assay

The CpG island on NR4A1 promoter was predicted by MethPrimer (http://www.urogene.org/cgi-bin/methprimer/methprimer.cgi). Genomic DNA was extracted from BC cells by using the QIAamp DNA Blood Mini Kit (Qiagen, cat. no. 51104), and bisulfite treatment was performed by using the EZ DNA Methylation-Gold Kit (Zymo Research, cat. no. D5005), following the manufacturer’s protocol. Then, the modified DNA was amplified, and PCR products were gel-purified and subcloned into a pESI-T vector system (Yeasen, cat. no. 10907). Ten colonies were sequenced to assess the degree of methylation at each CpG site by QUMA (http://quma.cdb.riken.jp). The primer sequences for bisulfite sequencing PCR were as follows: NR4A1, F 5′-GTTTTTTAGTTTGAGATTTTGTTGG-3′, R 5′-AAATCTCCTCACTCTCCAATTACTC-3′.

### Xenograft tumor model

To evaluate the influence of NR4A1 on BC growth, 8 × 10^6^ NR4A1-knockout or parental control MCF7 cells were suspended in 150 μl PBS and subcutaneously injected into the flank of 4–5-week-old BALB/c nu/nu female mice (*n* = 6 per group). For drug treatment assay, 8 × 10^6^ wild-type MCF7 cells were suspended in 150 μl PBS and subcutaneously injected into the flank of 4–5-week-old BALB/c nu/nu female mice. Seven days after subcutaneous inoculation, the mice were intraperitoneal injected with vehicle (dimethyl sulfoxide) or Csn-B (5 mg/kg) daily (*n* = 10 per group). All xenograft size was measured by (length × width^2^ × 0.5) every 3 days for 21 days. Mice were euthanized at the end of experiment, and xenograft tumors were extracted for analysis. Ki67 staining was performed for cell proliferative ability. All animal studies were conducted in accordance with protocols approved by Fujian Medical University Animal Care and Use Committee.

### Clinical specimens

The two tumor tissue microarrays (TMAs) used in this study were collected from Department of Thyroid and Breast Surgery, The First Affiliated Hospital of Fujian Medical University. The cohort 1 TMA contains 40 paired BC and normal breast tissues, and cohort 2 TMA contains 150 BC tissues. The pathological characteristics of each samples were examined by pathological specialists. This study was reviewed and approved by the Ethics Committees in The First Affiliated Hospital of Fujian Medical University with the written informed consents from all patients.

### Immunohistochemical analysis

The paraffin-embedded sections were dewaxed in xylene and rehydrated in alcohol. The sections were then exposed to antigen and incubated with a specific primary antibody against NR4A1 (Proteintech, cat. no. 12235-1-AP; 1:100 dilution), c-Fos (Abcam, cat. no. ab208942; 1:200 dilution) or PRDX6 (Abcam, cat. no. ab133348; 1:100 dilution) at 4 °C overnight, followed by incubating with the corresponding secondary antibodies at 37 °C for 1 h. Representative images were taken using an Olympus light microscope.

The staining results were measured by the semiquantitative scoring systems by two pathologists who were blinded to the clinical outcomes through immunoreactive scores (IRS), based on the staining intensity and area. In general, each specimen was assigned a score according to the intensity of staining (0, no staining; 1, weak staining; 2, moderate staining; 3, strong staining), and the percentage of stained cells (0, 0%; 1, 1–24%; 2, 25–49%; 3, 50–74%; 4, 75–100%). The final IRS was determined by multiplying the intensity score with the score for the percentage of stained cells. As a result, nine grades were scored as 0, 1, 2, 3, 4, 6, 8, 9 and 12. The staining pattern of NR4A1 was defined as low expression (IRS 0–4) and high expression (IRS 6–12).

### Statistical analysis

Data are expressed as the mean ± s.d. of three independent experiments and analyzed by the SPSS software program (version 22.0). Two-tailed unpaired or paired Student’s *t*-test was used for two-group comparisons, and one-way analysis of variance (ANOVA) test was used for three or more group comparisons. Chi-square test was utilized to analyze the correlation between NR4A1 expression and clinicopathologic features of patients with BC. Kaplan–Meier survival analysis and log-rank test were performed for survival rate calculation and comparison between indicated groups. Univariate and multivariate Cox proportional hazards model were used to evaluate the significance of NR4A1 and clinicopathological features on overall survival. Person correlation coefficient was performed to analyze the correlation between the IRS of immunohistochemistry (IHC) staining. *P* < 0.05 was considered statistically significant.

## Results

### NR4A1 expression is downregulated in BC tissues and is associated with cancer progression

**Fig. 1:**
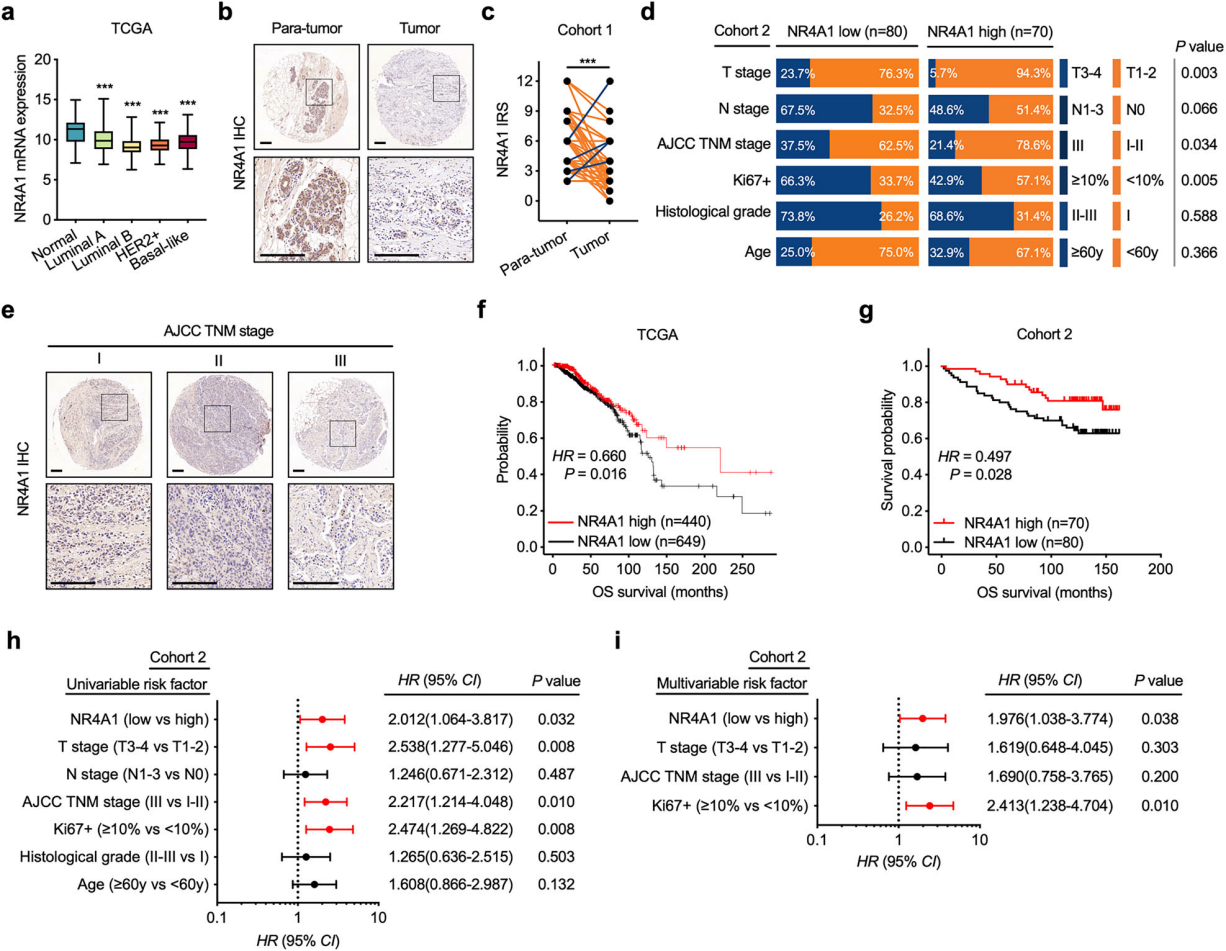
NR4A1 has a tumor-suppressive role in BC progression. **a** Analysis of the TCGA database shows that the NR4A1 mRNA level is decreased in all four types of BC samples compared with normal breast tissues. Unpaired Student’s *t*-test, ***P < 0.001. **b** Representative IHC staining of NR4A1 in cohort 1 TMA. Scale bars, 200 μm. **c** Quantitative IRS of NR4A1 as in **b**. Paired Student’s *t*-tests, ***P < 0.001. **d** Stratification of patients in cohort 2 with NR4A1 high expression (with IRS 6–12) or low expression (with IRS 0–4) at the protein level in TMA and its association with clinicopathological factors. Chi-square test. **e** IHC staining of NR4A1 in different AJCC TNM stages of BC. Scale bar, 200 μm. **f**, **g** Kaplan–Meier survival plots of patients with BC based on NR4A1 expression in TCGA cohort (**f**) and cohort 2 (**g**). OS, overall survival; HR, hazard ratio. **h**, **i** Log-rank tests. Univariate (**h**) and multivariate (**i**) analysis was performed in cohort 2. The bars correspond to 95% confidence interval (CI).

According to the The Cancer Genome Atlas (TCGA) and Molecular Taxonomy of Breast Cancer International Consortium (METABRIC) databases, NR4A1 mRNA was significantly decreased in all subtypes of BC (Fig. 1a). NR4A1 expression was also evaluated in a TMA with pairs of tumor and paratumor tissues from patients with BC (cohort 1). Similarly, NR4A1 expression was significantly lower in tumor tissues than in paired nontumor tissues (Fig. 1b, c). We subsequently used a second BC cohort (cohort 2) to analyze the associations between NR4A1 and BC patient clinicopathologic characteristics. No significant correlation was detected between NR4A1 expression and patient age, tumor histological grade or lymph node metastasis (Fig. 1d), but NR4A1 expression was negatively associated with the American Joint Committee on Cancer (AJCC) tumor stage, TNM stage and Ki67 proliferative index of patients with BC (Fig. 1d, e). Moreover, there was a positive correlation between NR4A1 expression and overall postsurgical survival in the TCGA BC cohort (Fig. 1f), which was further confirmed in our BC cohort (cohort 2; Fig. 1g). In addition, univariate (Fig. 1h) and multivariate (Fig. 1i) regression analyses of cohort 2 demonstrated that NR4A1 expression was an independent prognostic indicator of BC overall survival, with significant hazard ratios for predicting clinical outcomes. Together, these findings suggest that NR4A1 plays a tumor-suppressive role in BC.

Next, we investigated the potential mechanism that mediates the downregulated expression of NR4A1 in BC. DNA hypermethylation is one of the major mechanisms that directly induces gene transcription repression. An analysis of the DNA methylation profiles and transcription levels of NR4A1 from the BC tissues in the TCGA cohort revealed a statistically negative correlation between higher DNA methylation levels at the NR4A1 genomic locus and lower transcription levels of NR4A1 mRNA (Supplementary Fig. 1a). In addition, two CpG islands were identified in the promoter sequence of NR4A1 (Supplementary Fig. 1b), and the overall methylation frequency in the CpG islands of the NR4A1 promoter was significantly decreased by treatment with the DNA methylation inhibitor 5-azacytidine (Supplementary Fig. 1c), followed by increased NR4A1 mRNA levels in BC cells in a 5-azacytidine dose-dependent manner (Supplementary Fig. 1d). Together, these results suggest that the downregulated expression of NR4A1 mRNA in BC results from DNA hypermethylation.

### Deletion of NR4A1 promotes BC cell growth

**Fig. 2:**
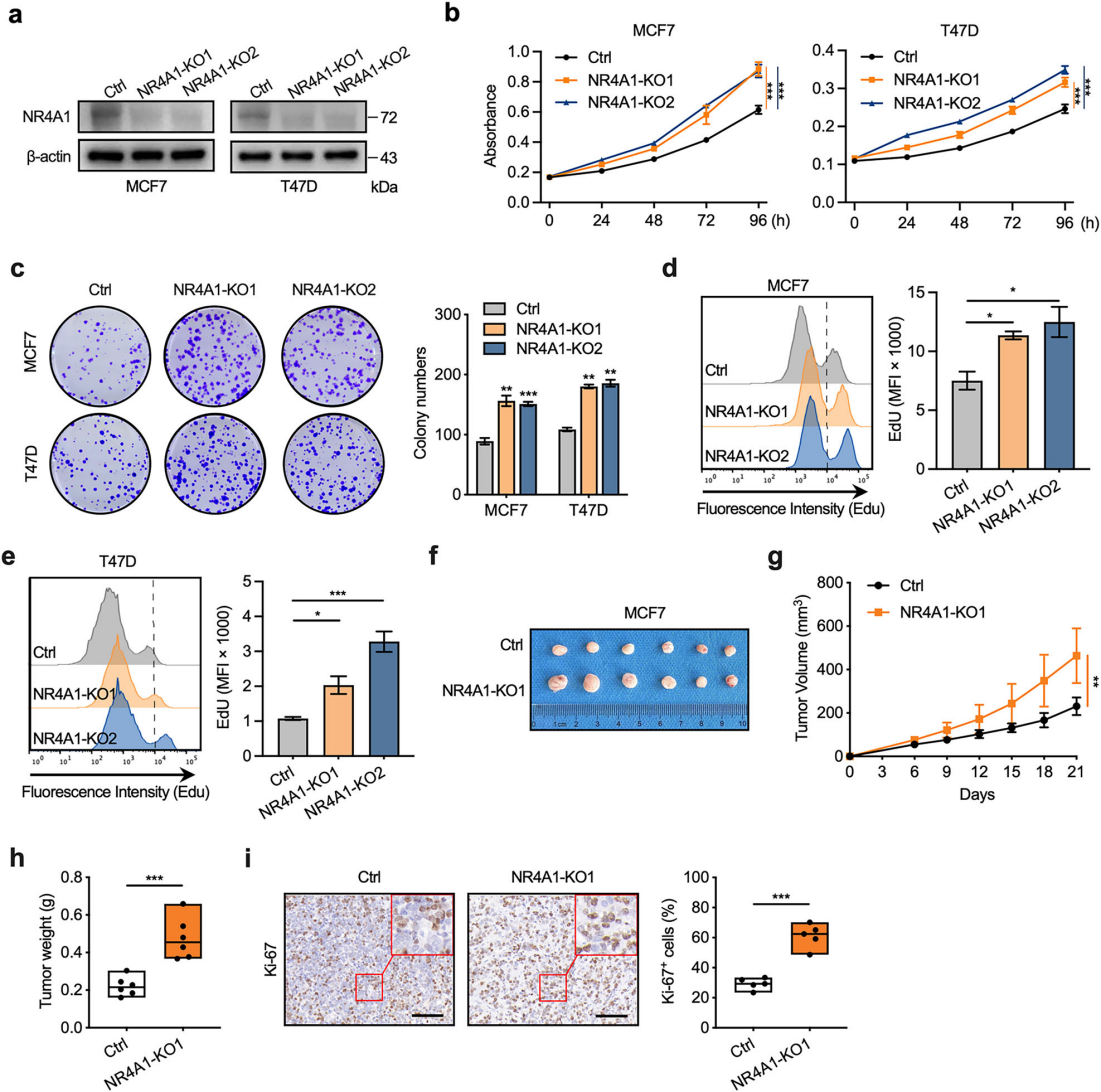
NR4A1 deficiency promotes the proliferation of BC cells. **a** Immunoblotting analysis of NR4A1 knockout efficiency in MCF7 and T47D cells. **b** Cell proliferation assay was performed by CCK-8 assay in NR4A1-knockout and parental control MCF7 cells or T47D cells. **c** Colony formation assay was performed in NR4A1-knockout and parental control MCF7 cells or T47D cells. **d**, **e** Flow cytometry analysis (left) and statistical quantification of MFI (right) for Edu incorporation in NR4A1-knockout and parental control MCF7 cells (**d**) or T47D cells (**e**). MFI, mean fluorescence intensity. **f**–**h** NR4A1-knockout and parental control MCF7 cells were injected into the flank of female athymic nude mice (*n* = 6 per group). Tumor volumes were measured every 3 days. Tumor images (**f**), growth curves (**g**) and tumor weight (**h**) were obtained at day 21 after dissection. **i** Representative IHC staining of Ki67 and the statistical analysis of Ki67-positive percentages in tumors from **f**. Scale bar, 100 μm. Unpaired Student’s *t*-tests were used in **c**–**e**, **h** and **i**, and one-way ANOVA was used in **b** and **g**, *P < 0.05, **P < 0.01, ***P < 0.001.

We then assessed the biological effect of NR4A1 in BC cells. Previous studies have shown that loss of NR4A1 increases the proliferative ability of basal-like and HER2-positive BC cells^24,34^, but the role of NR4A1 in luminal BC cells remains uncertain. Therefore, we further explored the biological effect of NR4A1 in luminal BC cells, including MCF7 and T47D cells. NR4A1 knockout was generated in MCF7 and T47D cells using the CRISPR–Cas9 approach (Fig. 2a). Knockout of NR4A1 promoted the proliferation of BC cells (Fig. 2b). Consistently, NR4A1 deletion effectively enhanced the ability of MCF7 and T47D cells to form colonies (Fig. 2c). By contrast, ectopic NR4A1 overexpression inhibited MCF7 and T47D cell growth (Supplementary Fig. 2a, b). In addition, endogenous NR4A1 deletion potently induced the incorporation of EdU (Fig. 2d, e). Moreover, in vivo studies revealed that, when propagated as xenografts in mice, MCF7 cells were substantially more tumorigenic in the absence of endogenous NR4A1 (Fig. 2f–h). IHC revealed that NR4A1 deletion increased Ki67 expression, indicating enhanced cell proliferation (Fig. 2i). To further investigate the tumor-suppressive role of NR4A1 in preventing the malignant transformation of normal mammary epithelial cells, the immortalized normal mammary epithelial cell line MCF10A was utilized. Consistent with previous findings that NR4A1 in mammary tissues inhibited mammary tumor development^25^, we found that NR4A1 knockout in MCF10A cells resulted in a more proliferative and aggressive phenotype (Supplementary Fig. 3a–c). Taken together, these data suggest that NR4A1 has antiproliferative activity in BC cells and antimalignant transformation ability in normal mammary cells.

### NR4A1 deficiency leads to altered lipid metabolism and phospholipid accumulation

**Fig. 3:**
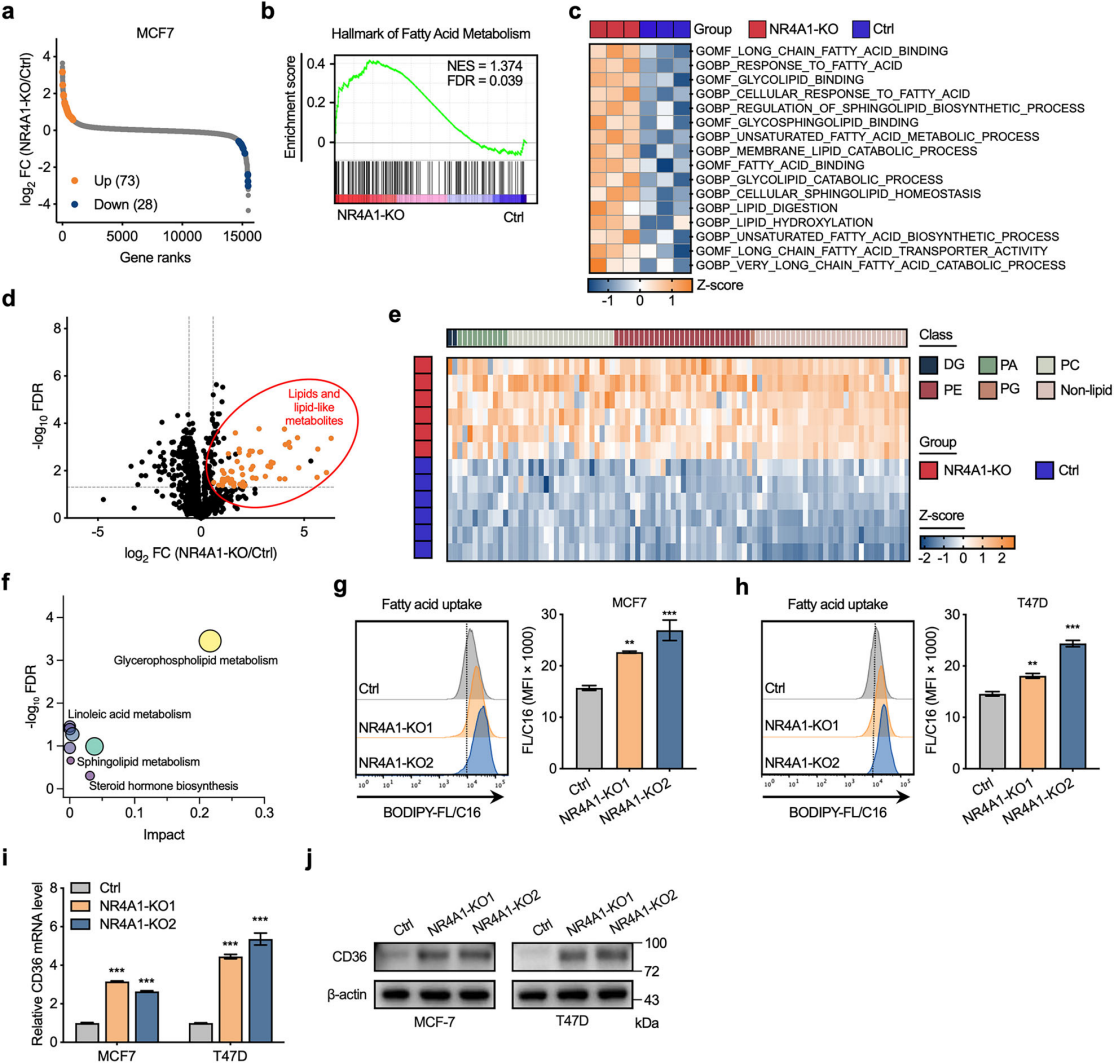
NR4A1 deficiency regulates the lipid remodeling and phospholipid accumulation. **a** Rank-ordered depiction of fold changes for all analyzed genes quantified by RNA-seq with the significantly changed genes of 73 increased and 28 decreased (fold change >1.5 and FDR <0.05 difference) in NR4A1-knockout MCF7 cells compared with parental MCF7 cells. **b** GSEA analysis was conducted to identify the different gene profiles between NR4A1-knockout and parental MCF7 cells. **c** Single-sample GSEA analysis was performed to show the pathways closely correlated with NR4A1 expression in MCF7 cells. **d** Volcano plots of metabolites detected by UHPLC–QTOF–MS-based nontargeted metabolomics analysis in NR4A1-knockout and parental MCF7 cells. Red represents lipids and lipid-like metabolites (*n* = 61). **e** Heat map showed the classification of the significantly changed metabolites of 91 increased (fold change >1.5, FDR <0.05) in NR4A1-knockout MCF7 cells compared with parental MCF7 cells. **f** Enriched metabolic signaling pathways based on significantly changed metabolites (*n* = 199) cluster identified by pathway analysis (https://www.metaboanalyst.ca/). **g**, **h** Flow cytometry analysis (left) and statistical quantification of MFI (right) for BODIPY FL C16 to compare fatty acid uptake ability in NR4A1-knockout and parental control MCF7 or T47D cells. **i**, **j** RT-qPCR (**i**) and immunoblotting analysis (**j**) of CD36 **i**n NR4A1-knockout and parental MCF7 or T47D cells. Unpaired Student’s *t*-tests were used in **g**–**i**, **P < 0.01, ***P < 0.001.

To elucidate which biological processes are altered by NR4A1 suppression of BC cell growth, we performed transcriptome analysis to compare the gene expression profiles of NR4A1-knockout and parental control cells. A total of 73 upregulated genes and 28 downregulated genes were detected in NR4A1-knockout cells (Fig. 3a and Supplementary Fig. 4a). GSEA of the transcription profiles revealed that the gene signature of ‘fatty acid metabolism’ was enriched in NR4A1-knockout cells (Fig. 3b). In addition, single-sample GSEA confirmed that lipid and lipid-related processes were positively correlated with NR4A1-knockout cells (Fig. 3c).

Cancer cells are known to exhibit greater metabolic plasticity than normal cells. Studies have shown that cancer cells rewire their metabolism to support the biosynthetic requirements of uncontrolled proliferation, rapid growth and long-term maintenance^35–37^. To determine the role of NR4A1 in cancer metabolism, we performed a comprehensive untargeted mass spectrometric metabolomics analysis in NR4A1-knockout and parental control BC cells. Compared with those in the control cells, 199 metabolites were significantly altered upon NR4A1 deletion (Fig. 3d and Supplementary Table 1). The upregulated metabolites (61 out of 91) were mostly lipids and lipid-like metabolites (Fig. 3d), which mainly included PE, phosphatidylcholine (PC), phosphatidic acid (PA) and phosphatidyl glycerol (PG) (Fig. 3e). As phospholipids are major constituents of biological membranes, a greater abundance of phospholipids in cancer cells impacts cell proliferation^38^. Further pathway enrichment analysis of the differentially abundant metabolites also indicated that the metabolism of glycerophospholipids, linoleic acid and sphingolipids was enriched in the NR4A1-knockout cells (Fig. 3f). These results suggest that the contents and characteristics of the lipidome in BC cells are notably altered by NR4A1 knockout.

Furthermore, by using a fluorescent analog of palmitic acid (BODIPY FL C16) staining assay, we found that NR4A1-knockout BC cells presented increased lipid uptake (Fig. 3g, h). We then assessed the expression of CD36, which is one of the most important major fatty acid receptors that participates in BC fatty acid uptake^39,40^. Higher expression of CD36 was detected in NR4A1-knockout BC cells (Fig. 3i, j and Supplementary Fig. 4a), which was consistent with the findings of a previous report^25^. Taken together, these results provide evidence that NR4A1 controls lipid remodeling to regulate BC cell growth.

### NR4A1 deficiency promotes increased levels of oxidative stress

**Fig. 4:**
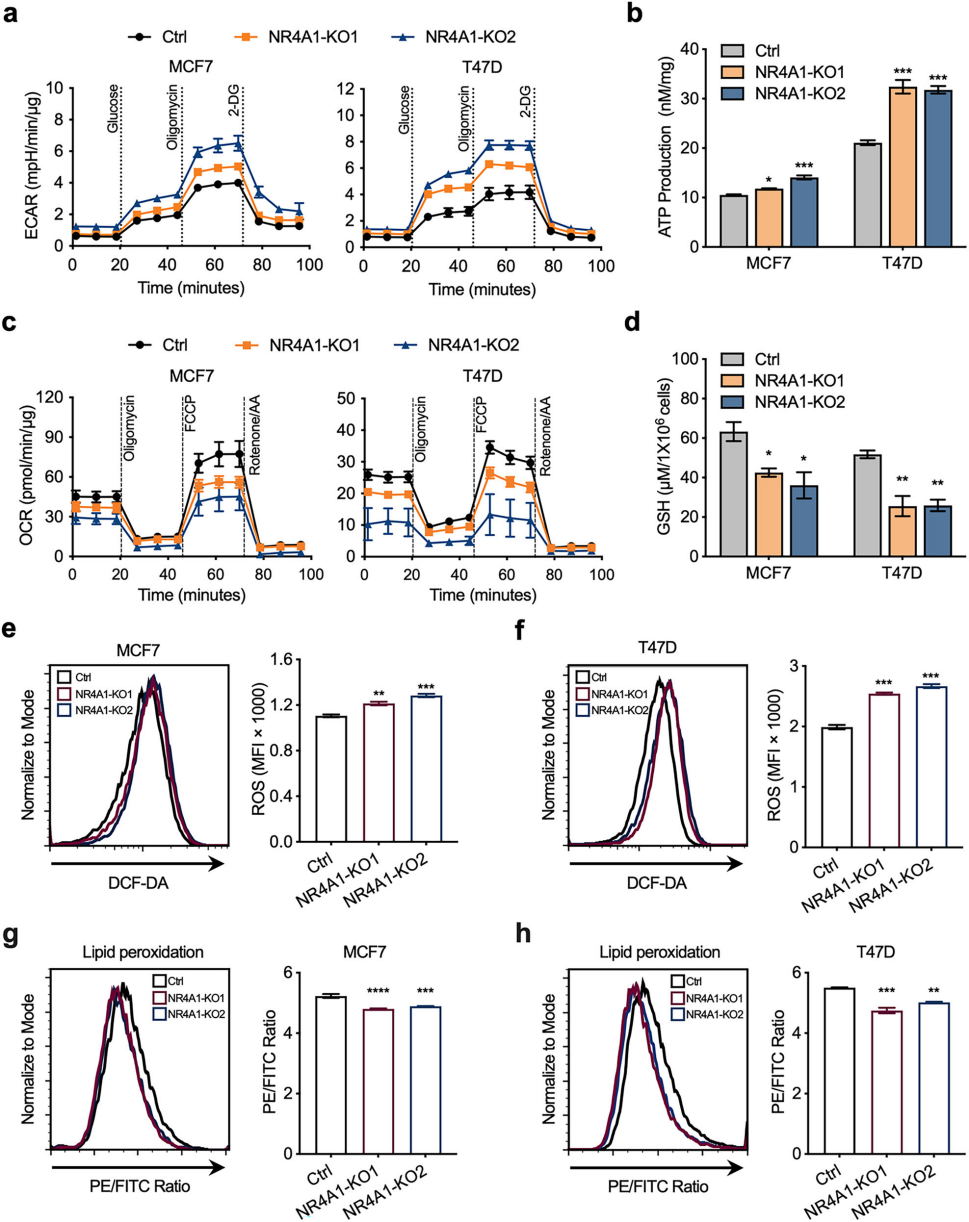
Suppression of NR4A1 exacerbates the redox balance disruption. **a** Seahorse extracellular flux analyzer measurement of ECAR metabolic profile in NR4A1-knockout and parental MCF7 or T47D cells. **b** Assessments of ATP production ability in NR4A1-knockout and parental MCF7 or T47D cells. **c** Seahorse extracellular flux analyzer measurement of OCR metabolic profile in NR4A1-knockout and parental MCF7 or T47D cells. **d** Intracellular GSH level in MCF7 and T47D cells with or without NR4A1 expression. **e**, **f** Flow cytometry analysis of intracellular ROS levels by DCFH-DA staining in NR4A1-knockout and parental MCF7 (**e**) or T47D (**f**) cells. ROS levels were quantified by MFI. **g**, **h** Lipid peroxidation measured by flow cytometry using the lipid peroxidation reagent in NR4A1-knockout and parental MCF7 (**g**) or T47D (**h**) cells. Lipid peroxidation was quantified by the ratio of red (PE)/green (FITC) fluorescence intensities, and the decreased ratio was correlated with higher lipid peroxidation. Unpaired Student’s *t*-tests were used in **b** and **d**–**h**, *P < 0.05, **P < 0.01, ***P < 0.001.

To further characterize the metabolic effects of NR4A1 in BC, we performed metabolic profiling using a Seahorse instrument. NR4A1 deletion significantly increased the ECAR, reflecting an increase in glucose flux (Fig. 4a). In addition, the intracellular ATP levels in the NR4A1-knockout cells were higher than those in the parental control cells (Fig. 4b). Suppression of NR4A1 could also increase the glycolytic capacity through a mechanism that is reliant on elevated cellular lipid levels. However, the OCR was disrupted upon NR4A1 deletion, with a significant decrease in basal and maximal respiration (Fig. 4c). Moreover, the absence of NR4A1 resulted in decreased GSH levels (Fig. 4d) and increased ROS production (Fig. 4e, f). The accumulation of oxidative stress further caused significant lipid peroxidation inside the NR4A1-knockout cells (Fig. 4g, h), suggesting that NR4A1 had a multifaceted effect on lipid remodeling and cell redox homeostasis. High levels of oxidative stress can accelerate the growth and development of BC^41^. Together, these results indicate that redox balance disruption exacerbated by NR4A1 deficiency is involved in uncontrolled tumor growth.

### NR4A1 interacts with c-Fos to regulate its transcriptional activity

**Fig. 5:**
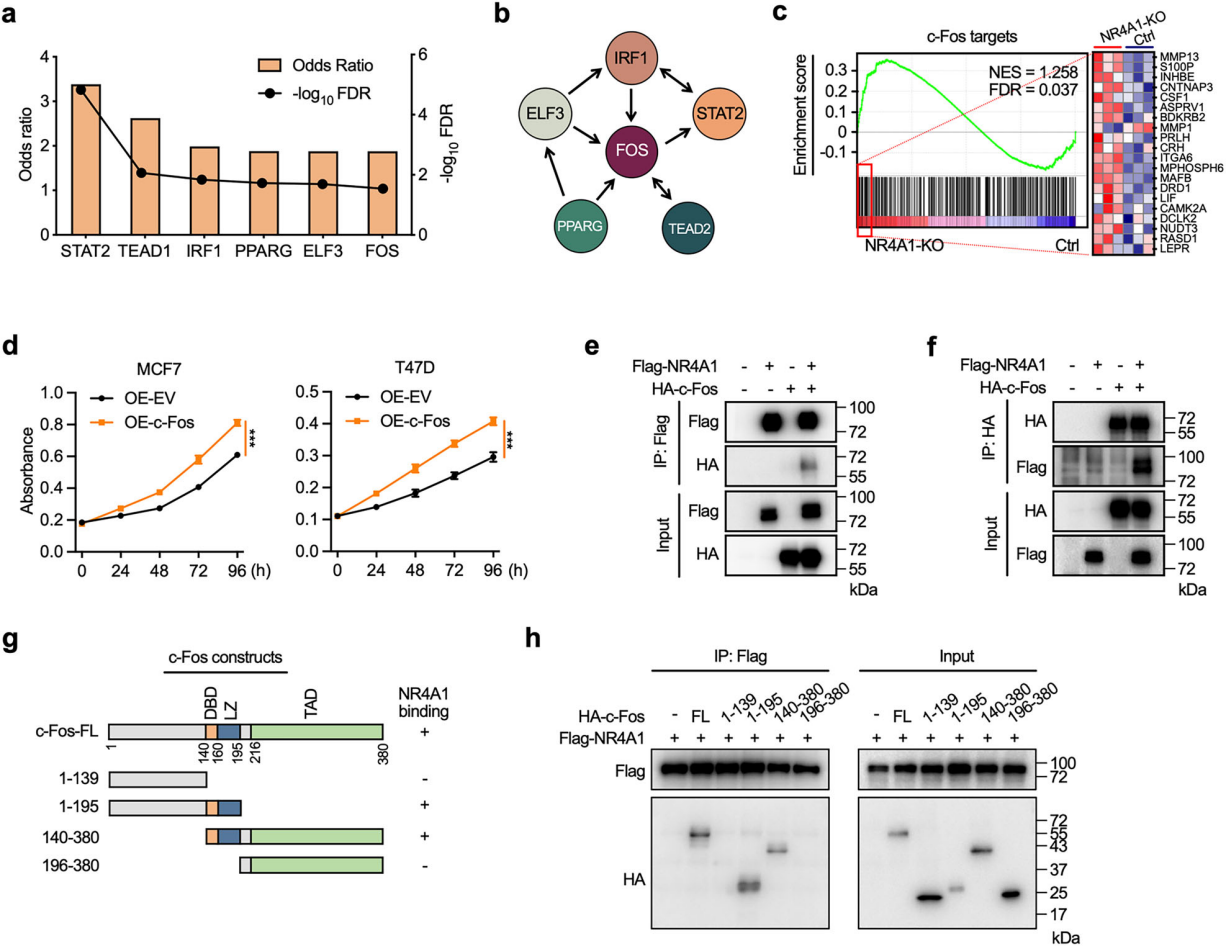
NR4A1–c-Fos interaction affects the transcriptional activity of c-Fos. **a**, **b** Prediction of transcription factors regulating the significantly upregulated genes in NR4A1-knockout MCF7 cells by using TFEA method in ChEA3 databases. Statistical results (**a**) and co-regulatory network for the functional interaction (**b**) for the top six predicted transcription factors. **c** Enrichment plot of the c-Fos dataset in GSEA analysis. Heat map of the top 20 genes upregulated in NR4A1-knockout cells from the c-Fos dataset. **d** Cell proliferation assay was performed by CCK-8 assay in ectopic c-Fos- or empty vector (EV)-overexpressed MCF7 cells or T47D cells. One-way ANOVA, ***P < 0.001. **e**, **f** Co-IP experiments to detect the interaction between NR4A1 and c-Fos. HEK293T cells were transfected for 24 h with plasmids encoding either Flag-NR4A1 or HA-c-Fos alone or in combination. Cell lysates were immunoprecipitated with Flag (**e**) or HA (**f**) antibodies. **g** c-Fos domain structure and deletion mutants used for Co-IP experiments. **h** Co-IP experiments were used to identify the interaction domain for c-Fos binding to NR4A1.

We next sought to address the biological mechanism underlying the disruption of lipid metabolism and redox homeostasis upon NR4A1 deficiency. TFEA of differentially expressed genes in NR4A1-knockout cells revealed that the transcription factor FOS-associated transcriptome was one of the most upregulated transcriptional programs and functionally interacted and overlapped with other predicted transcription-factor-mediated transcription activation (Fig. 5a, b). In addition, GSEA revealed that c-Fos (FOS gene-encoded protein) target genes, including lipid metabolism-related genes, such as MMP1, LIF, CAMK2A and LEPR, were significantly overexpressed in NR4A1-knockout BC cells compared with parental control cells (Fig. 5c), which were validated by RT-qPCR in luminal, basal-like and HER2-positive BC cells (Supplementary Fig. 5a, b). This finding implies that c-Fos might act as the chief mediator in uncontrolled tumor growth following NR4A1 deficiency. Moreover, consistent with the accelerated cell proliferation following NR4A1 knockout, ectopic c-Fos overexpression promoted BC cell growth (Fig. 5d).

c-Fos, a core member of the activator protein-1 (AP-1) family of transcription factors, acts as an oncogenic factor in multiple cancers^42–44^. Growing evidence shows that c-Fos is involved in the initiation and progression of BC^45–47^. To determine the functional relevance between NR4A1 and c-Fos, we sought to uncover the precise regulatory mechanism involved. Co-IP assays verified that the NR4A1 protein could interact with the c-Fos protein (Fig. 5e, f). Considering that c-Fos is largely thought of as a transcription factor based on genome binding, we further examined whether the formation of the NR4A1–c-Fos interaction affects the ability of c-Fos to bind chromatin. We mapped the domains in c-Fos that mediated their interactions. The c-Fos protein consists of a DNA-binding domain (amino acids 140–160) for chromatin binding, a leucine zipper (amino acids 161–195) for heterodimerizing and consequently binding to DNA, and an unstructured transcriptional activation domain at the C-terminus (Fig. 5g). Deletion analysis indicated that NR4A1 interacted with c-Fos in the c-Fos DNA-binding domain/leucine zipper domain, which is needed for c-Fos binding to the genome (Fig. 5h). These data suggest that the increase in lipid remodeling-associated gene transcription after NR4A1 deletion might be a result of the loss of the interaction between NR4A1 and c-Fos, followed by enhanced c-Fos-mediated transcription.

### NR4A1 competitively inhibits c-Fos binding to target genes

**Fig. 6:**
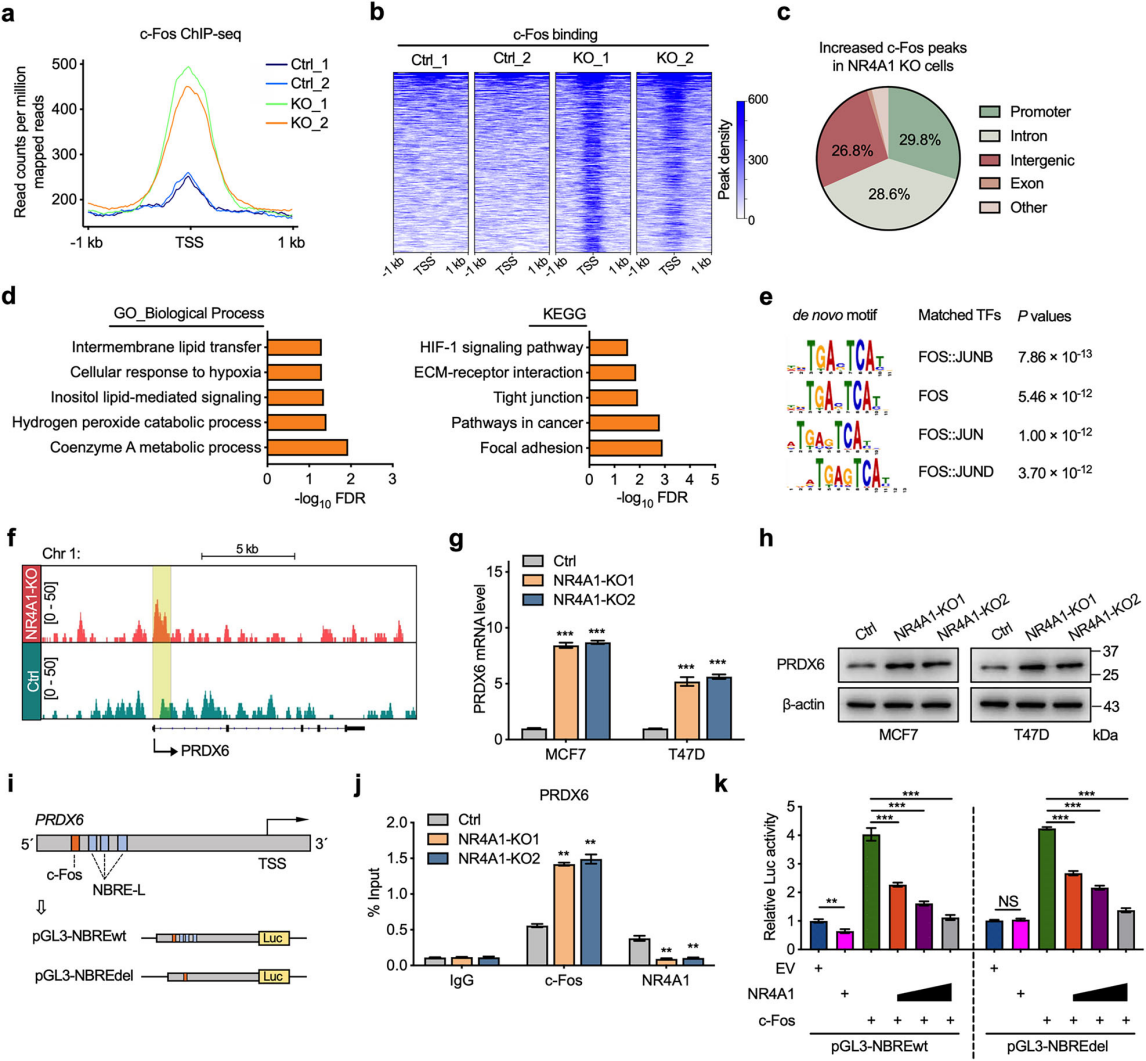
NR4A1 competitively inhibits c-Fos binding to targeted genes in BC cells. **a** Genome-wide profiles of c-Fos in NR4A1-knockout and control MCF7 cells. **b** Heat maps of c-Fos ChIP–seq signals sorted on the basis of increased c-Fos peaks between NR4A1-knockout and control MCF7 cells. **c** Genomic annotations of the increased c-Fos peaks in NR4A1-knockout cells by chromosome location. **d** GO analysis and KEGG analysis of NR4A1-competitive c-Fos occupied genes. **e** Motif sequences (left) and matched transcription factors (middle) with corresponding *P* values (right) from de novo motif analysis of NR4A1-competitive c-Fos peaks. **f** c-Fos ChIP–seq tracks at PRDX6 gene locus. **g** RT-qPCR analysis of PRDX6 mRNA levels in NR4A1-knockout and control cells. **h** Immunoblotting analysis of PRDX6 in BC cells with or without NR4A1. **i** Schematic diagram of PRDX6 promoter showing c-Fos and NR4A1 binding motifs in the regulator region. pGL3-NBREwt and pGL3-NBREdel stand for the PRDX6 promoter region with NBRE-like elements or NBRE-like elements deleted sequences, which were cloned upstream of the firefly luciferase gene in the pGL3-basic vector. **j** ChIP–qPCR analysis of c-Fos and NR4A1 enrichment around the c-Fos motif and NBRE-like elements on PRDX6 promoter in NR4A1 knockout and control MCF7 cells. **k** PRDX6 promoter constructs were co-transfected with NR4A1, c-Fos or empty vector (EV) to detect luciferase activity in HEK293T cells. pRL-TK was transfected for normalization, and luciferase activity was measured by using a dual luciferase reporter assay system. Unpaired Student’s *t*-tests were used in **g**, **j** and **k**, **P < 0.01, ***P < 0.001, NS nonsignificant.

We hypothesized that NR4A1 competes with c-Fos to prevent c-Fos from binding to the genome. c-Fos signals from ChIP–seq analysis demonstrated a genome-wide increase after NR4A1 knockout in MCF7 cells (Fig. 6a). A total of 373 increased c-Fos peaks were observed after NR4A1 knockout (Fig. 6b), including the CD36 genome locus (Supplementary Fig. 6a). Specifically, 29.8% of the increased c-Fos peaks caused by NR4A1 knockout were located mainly at promoters (Fig. 6c). The signaling pathways related to lipid metabolism, oxidation and oncogenesis were enriched in the increased c-Fos peak-located genes (Fig. 6d). In addition, the increased c-Fos peaks caused by NR4A1 depletion were enriched in binding to AP-1 families, practically FOS targets (Fig. 6e), suggesting that these increased c-Fos peaks caused by NR4A1 depletion occurred in an NR4A1-competitive manner. Moreover, the increased c-Fos peaks in NR4A1-expressing cells were also located at promoters and intron sequences, which might occur in an NR4A1-noncompetitive manner (Supplementary Fig. 6b, c). Together with our finding that NR4A1 interacts with the DNA-binding domains of c-Fos, these results confirm the hypothesis that NR4A1 interacts with c-Fos to interfere with its genome binding, especially in the promoter region, to repress gene transcription and maintain lipid and redox homeostasis in BC cells.

To identify the potential target of NR4A1-competitive c-Fos peaks that could lead to disrupted lipid metabolism and redox homeostasis in NR4A1-knockout cells, we screened the NR4A1-competitive c-Fos peak-containing genes. Interestingly, we found that the c-Fos signal at the promoter locus of peroxiredoxin 6 (PRDX6) was significantly enriched in the NR4A1-knockout cells compared with the parental control cells (Fig. 6f). Furthermore, the deletion of NR4A1 upregulated the expression of PRDX6 in MCF7 and T47D cells at the mRNA level, followed by an increase in the protein level (Fig. 6g, h). A consistent increase in PRDX6 mRNA levels caused by NR4A1 deletion was also observed in basal-like cells and HER2-positive BC cells (Supplementary Fig. 6d).

As a bifunctional protein, in addition to its role in redox balance, PRDX6 harbors both Ca^2+^-independent phospholipase A2 (iPLA2) and lysophosphatidylcholine acyltransferase (LPCAT) activities, which participate in tumor phospholipid remodeling to promote abnormal growth^48^. PRDX6 was previously described as an oncogene during the progression of BC^49,50^. Thus, we further validated the mechanism by which NR4A1/c-Fos controls PRDX6 transcription. Here, sequence analysis revealed a putative c-Fos motif and three NBRE-like elements (NR4A1 motifs) within the promoter sequence of PRDX6 (Fig. 6i). Consistent with the NR4A1-competitive c-Fos peak at the PRDX6 genome locus, the occupancy of c-Fos at the promoter of PRDX6 was significantly greater in NR4A1-knockout cells than in parental control cells (Fig. 6j and Supplementary Fig. 6e). In addition, NR4A1 could recognize and access NBRE-like elements within the PRDX6 promoter region (Fig. 6j). As validated by luciferase assays, c-Fos activated and NR4A1 inhibited PRDX6 promoter activity (Fig. 6k).

To further reveal the competitive role of c-Fos and NR4A1 in the transcriptional regulation of PRDX6, we generated a mutant reporter that lacked the NBRE-like elements in the PRDX6 promoter (Fig. 6i). NBRE-like element deletion abolished the NR4A1-mediated repression of the PRDX6 promoter but barely reversed the effect of c-Fos overexpression on PRDX6 promoter activity (Fig. 6k). Surprisingly, although the ectopic expression of NR4A1 could not inhibit the transcriptional activity of the NBRE-deleted PRDX6 promoter, NR4A1 could still diminish the transcriptional activity of the NBRE-deleted PRDX6 promoter in the presence of c-Fos in an NR4A1 abundance-dependent manner (Fig. 6k). Next, we performed in vivo analysis to further investigate the competitive role of c-Fos and NR4A1 in BC cells. We generated genome-edited MCF7 cells using a CRISPR–Cas9 approach in which the NBRE-like elements in the PRDX6 promoter were deleted (Supplementary Fig. 7a). We found that this deletion could abrogate NR4A1 binding to the PRDX6 promoter but could not prevent c-Fos binding (Supplementary Fig. 7b). Notably, knockdown of endogenous NR4A1 in NBRE-like element-deleted MCF7 cells increased c-Fos binding to the PRDX6 promoter, followed by the activated transcription of PRDX6; this was in contrast to the finding that NBRE-like element-deleted MCF7 cells with endogenous NR4A1 expression (Supplementary Fig. 7b, c), which suggested that NR4A1 inhibits c-Fos-mediated transcription activation independent of NR4A1 genome binding. Collectively, these results demonstrated that NR4A1 serves as an essential effector that competes with c-Fos to inhibit the transcription of PRDX6 to restrain lipid remodeling and redox dyshomeostasis in BC cells.

### Activation of NR4A1 inhibits the transcriptional activity of c-Fos

**Fig. 7:**
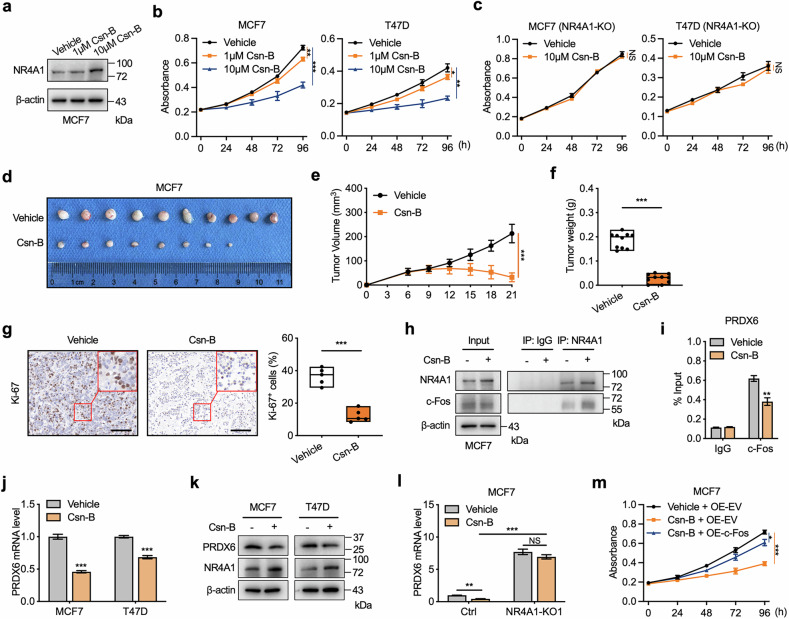
NR4A1 agonist represses the transcriptional activity of c-Fos. **a** Immunoblotting analysis of NR4A1 in MCF7 cells treated by different concentrations of Csn-B or vehicle. **b** Cell proliferation assay was performed by CCK-8 assay in MCF7 or T47D cells treated by different concentrations of Csn-B or vehicle. **c** Comparison of cell growth between Csn-B (10 μM) and vehicle treatment in NR4A1-knockout MCF7 cells or T47D cells. **d**–**f**, Csn-B inhibited BC growth in xenografts. Female athymic nude mice bearing wild-type MCF7 tumor were treated daily with vehicle or Csn-B. Tumor images (**d**), growth curves (**e**) and tumor weight (**f**) were obtained at the end of treatment. *n* = 10 per group. **g** Representative IHC staining of Ki67 and the statistical analysis of Ki67-positive percentages in tumors **d**. Scale bar, 100 μm. **h** Co-IP experiments to detect interactions between NR4A1 and c-Fos in MCF7 cells treated by 10 μM Csn-B or vehicle for 24 h. **i** ChIP assays of the c-Fos enrichment on PRDX6 promoter in MCF7 cells treated by 10 μM Csn-B or vehicle for 24 h. **j**, **k** RT-qPCR (**j**) and immunoblotting analysis (**k**) of PRDX6 in MCF7 or T47D cells treated by 10 μM Csn-B or vehicle for 24 h. **l** Comparison of PRDX6 mRNA levels in NR4A1-knockout and parental MCF7 cells treated by 10 μM Csn-B or vehicle for 24 h. **m** Cell proliferation assay was performed by CCK-8 assay in ectopic c-Fos- or EV-overexpressed MCF7 cells treated by 10 μM Csn-B or vehicle. Unpaired Student’s *t*-tests were used in **f**, **g**, **i**, **j** and **l**, and one-way ANOVA was used in **b**, **c**, **e** and **m**, *P < 0.05, **P < 0.01, ***P < 0.001, NS nonsignificant.

The results above confirmed the tumor-suppressive role of NR4A1 in BC growth, suggesting that restoring the activity or expression of NR4A1 might be a therapeutic strategy for inhibiting BC growth. To determine whether pharmacological activation of NR4A1 inhibits the growth of BC cells, we treated cells with a small-molecule agonist, cytosporone B (Csn-B), which specifically binds to the ligand-binding domain of NR4A1 and stimulates NR4A1-dependent transcription of target genes, including NR4A1 itself^51^. As expected, Csn-B treatment increased the expression of NR4A1 at the transcriptional and protein levels (Fig. 7a and Supplementary Fig. 8a, b), followed by the inhibited growth of BC cells in a dose-dependent manner (Fig. 7b). However, the inhibitory effect of Csn-B on the growth of BC cells was abolished by NR4A1 knockout (Fig. 7c). Next, we performed in vivo analysis to confirm the pharmacological activation of NR4A1 in inhibiting BC growth in xenograft tumor models. Compared with vehicle treatment, daily injection of Csn-B in athymic nude female mice bearing MCF7 xenografts over a 2-week period significantly inhibited tumor growth (Fig. 7d–g). These results suggest that Csn-B inhibits BC growth both in vitro and in vivo by NR4A1 activation.

To clarify whether NR4A1 activation inhibits the growth of BC cells by interrupting c-Fos genome binding, we evaluated the interaction between NR4A1 and c-Fos under Csn-B treatment. Although Csn-B-induced NR4A1 did not affect c-Fos expression, Csn-B treatment of MCF7 cells increased NR4A1 protein expression, resulting in increased interaction abundance between NR4A1 and c-Fos (Fig. 7h). Furthermore, Csn-B treatment decreased the binding of c-Fos to the NR4A1-competitive c-Fos peak on the promoter of PRDX6 (Fig. 7i), which led to reduced transcription of PRDX6 (Fig. 7j, k and Supplementary Fig. 8c). In addition, NR4A1 deletion significantly diminished the inhibition of c-Fos-targeted PRDX6 transcription by Csn-B (Fig. 7l). Moreover, the overexpression of c-Fos partially reversed Csn-B-mediated growth arrest in BC cells (Fig. 7m). These results confirm that the pharmacological activation of NR4A1 inhibits the growth of BC cells by increasing the interaction between NR4A1 and c-Fos to impede the genome binding of c-Fos to its target genes.

### The NR4A1–c-Fos–PRDX6 axis contributes to BC prognosis

**Fig. 8:**
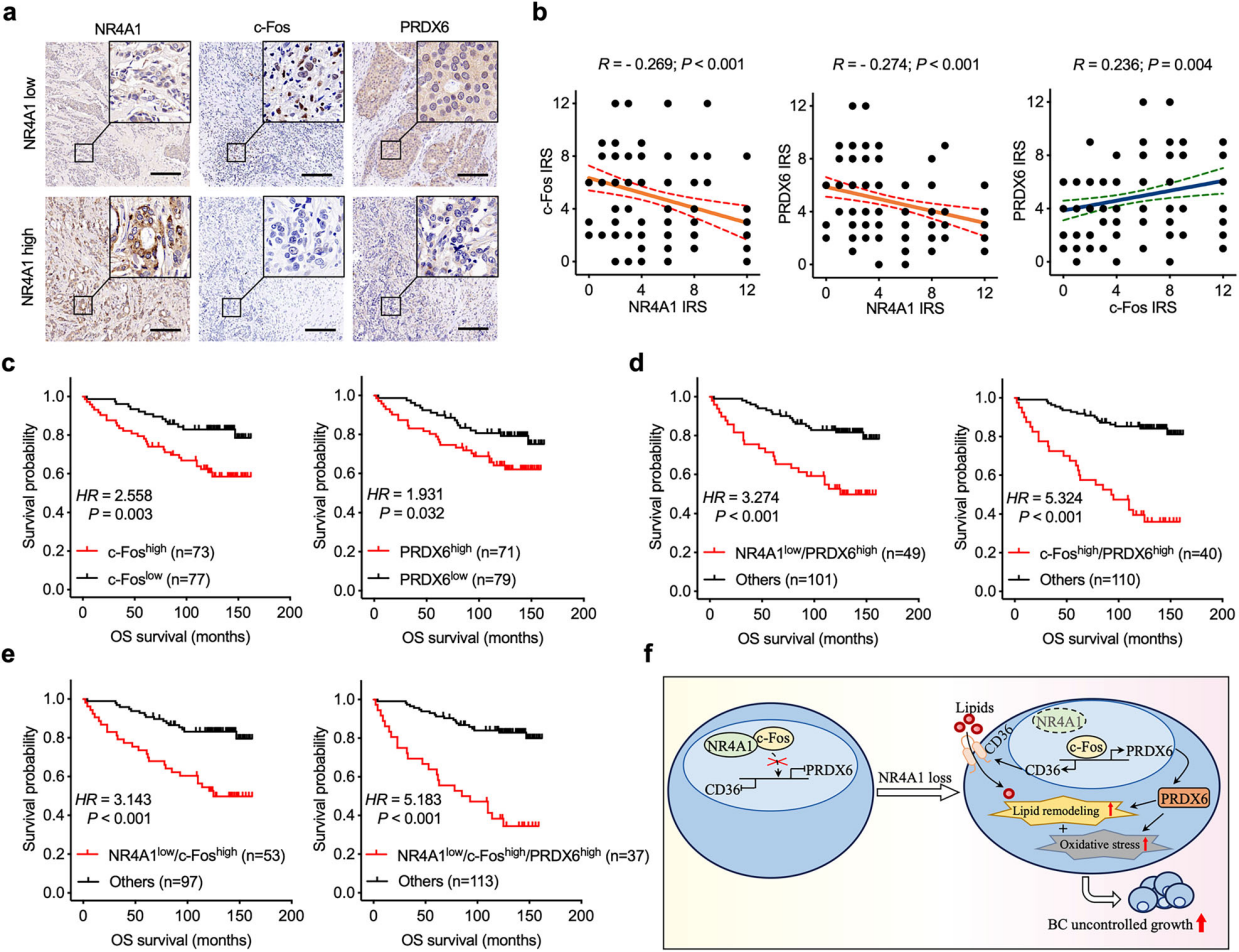
The impact of NR4A1–c-Fos–PRDX6 axis on BC prognosis. **a** Representative IHC staining of NR4A1, c-Fos and PRDX6 in cohort 2 TMA. Scale bars, 200 μm. **b** Pearson correlation analysis between NR4A1, c-Fos and PRDX6 protein expression based on IRS in cohort 2 TMA. *R* represents the Pearson correlation coefficient. **c** Kaplan–Meier survival plots of patients with BC in cohort 2 TMA based on c-Fos, or PRDX6 expression. **d** Kaplan–Meier survival plots of patients with BC in cohort 2 TMA based on the protein levels of NR4A1 co-expressed with PRDX6, or c-Fos co-expressed with PRDX6. **e** Kaplan–Meier survival plots of patients with BC in cohort 2 TMA based on the protein levels of NR4A1 co-expressed with c-Fos, or NR4A1 co-expressed with c-Fos and PRDX6. Log-rank tests were used in **c**–**e**. **f** Schematic diagram of the proposed mechanism.

To investigate the relationships among NR4A1, c-Fos and PRDX6 and human BC prognosis, we analyzed the expression of NR4A1, c-Fos and PRDX6 in the TMA of BC samples (cohort 2) (Fig. 8a) and found that NR4A1 expression was negatively correlated with c-Fos or PRDX6 expression (Fig. 8b). In addition, c-Fos was positively correlated with PRDX6 (Fig. 8b). We also confirmed that lower NR4A1 expression was correlated with poor prognosis in patients with BC (Fig. 1g); hence, we further investigated the prognostic value of c-Fos and PRDX6 in patients with BC. Patients with BC with higher expression of c-Fos or PRDX6 had much shorter overall survival than patients with lower expression of c-Fos or PRDX6 (Fig. 8c). Furthermore, patients with low levels of NR4A1 and high levels of PRDX6 or simultaneously high levels of c-Fos and PRDX6 had significantly shorter overall survival (Fig. 8d). Furthermore, we analyzed the triple correlation of NR4A1/c-Fos/PRDX6 and found that simultaneous low expression of NR4A1 and high expression of c-Fos and PRDX6 were associated with shorter overall survival in patients with BC (Fig. 8e).

Finally, we explored the underlying mechanism that contributes to the negative correlations between NR4A1 and c-Fos (Fig. 8b). Given that pharmacologic activation of NR4A1 did not affect c-Fos expression (Fig. 7h), we hypothesized that c-Fos might be an upstream regulator of NR4A1 expression. To test this hypothesis, we evaluated the effect of c-Fos in regulating NR4A1 expression. We found that ectopic c-Fos overexpression inhibited NR4A1 transcription at the mRNA level (Supplementary Fig. 9a). In addition, multiple consecutive c-Fos binding sites within CpG islands on the NR4A1 promoter were identified (Supplementary Fig. 9b). Hence, we generated a deletion mutant reporter that lacked the c-Fos binding sites on the NR4A1 promoter. A luciferase assay confirmed that this deletion mutation interfered with the ability of c-Fos to repress NR4A1 promoter activity (Supplementary Fig. 9c). Because the c-Fos binding site is located mainly within the CpG islands of the NR4A1 promoter and NR4A1 transcription is repressed by DNA hypermethylation, we further tested whether c-Fos could crosstalk with DNA methylation to repress NR4A1 transcription. A ChIP–qPCR assay confirmed that the DNA hypermethylation signal 5mC on the NR4A1 promoter was significantly increased by c-Fos overexpression (Supplementary Fig. 9d). Together, these results provide evidence that c-Fos cooperates with DNA hypermethylation to inhibit NR4A1 expression in BC (Supplementary Fig. 9e).

In summary, we revealed the pathogenic relationships among NR4A1, c-Fos and PRDX6 in patients with BC and demonstrated a functional network in which the NR4A1–c-Fos interaction inhibits c-Fos-mediated transcriptional control of lipid remodeling and redox dyshomeostasis in BC (Fig. 8f). Taken together, these findings show that the NR4A1–c-Fos–PRDX6 axis is a strong regulator of BC progression.

## Discussion

As BC is the leading cause of cancer mortality in women, the identification of additional mechanisms that participate in the progression of BC is essential for clinical applications. Here, we present NR4A1 as a key determinant that modulates BC growth by competing with c-Fos to regulate the transcription of PRDX6 and restrain lipid remodeling and redox balance disruption through comprehensive genomic, transcriptomic and metabolomic analyses.

As a transcription factor, the orphan nuclear receptor NR4A1 plays an oncogenic or tumor-suppressive role in tumor initiation and progression in a tumor-specific manner. Although NR4A1 is highly expressed in normal breast tissues but is decreased in tumorous breast tissues, the biological function of NR4A1 in BC has not been clearly explained, and it remains controversial whether NR4A1 has tumor-suppressive or tumor-promoting activity. A previous study revealed the suppressive role of NR4A1 in BC progression, as the loss of NR4A1 in mammary tissues accelerated the initiation of mammary tumors in a mouse model^25^. In our present work, NR4A1 deletion consistently increased the rapid proliferation and metastatic ability of immortalized normal mammary epithelial cells. In addition, loss of NR4A1 inhibits the growth of breast progenitor cancer cells^52^. Together, these findings provide evidence that NR4A1 expression in normal mammary cells could be protective against malignant development. The occurrence of malignant tumors is always accompanied by the rapid proliferation and invasion of tumor cells. Among these processes, the loss of NR4A1 enhances the proliferative ability of luminal BC cells, which is consistent with the suppressive role of NR4A1 in inhibiting basal-like and HER2-positive BC cells^24,34^, confirming the tumor-suppressive role of NR4A1 in the inhibition of BC growth. Contrary to these findings, NR4A1 has also been reported to promote the tumor metastasis of BC^23^. Therefore, the function of NR4A1 in BC might involve a cellular process-specific mechanism in which the loss of NR4A1 contributes to the malignant development of normal mammary cells, followed by the rapid proliferation of BC cells; however, the loss of NR4A1 could inhibit the metastatic ability of malignantly transformed BC cells. The underlying mechanisms for the opposing roles of NR4A1 in different cellular processes of BC need to be further investigated.

Cancer cells require amino acids, nucleotides and lipids for rapid proliferation. The accumulation or consumption of specific metabolites is associated with cellular metabolic rewiring, which is indispensable for the survival of cancer cells. Using the metabolomics analysis, we observed that lipid metabolism was a critically altered pathway in BC cells lacking NR4A1, along with marked phospholipid accumulation. The elevated level of the fatty acid transporter protein CD36 and increased lipid uptake further confirmed that lipid metabolism was remodeled by NR4A1 deficiency. Indeed, increasing evidence suggests that lipids participate in various biological processes in BC cells, including the synthesis of biological membrane phospholipids, signal transduction and energy sources^53–55^. For example, alterations in lipid metabolism and composition mediated by the loss of chromosome 8p promoted BC cell growth^56^. Furthermore, a recent report revealed that an altered lipid metabolism state is associated with the metastatic potential of the brain in basal-like BC^57^. In the present study, our results indicated the overlooked impact of NR4A1 on cancer cell metabolism.

The main precursor for fatty acid synthesis is glucose^58^. In this study, increased glucose utilization was observed in BC cells upon NR4A1 deletion via metabolic flux analysis, which showed the increased metabolic plasticity of BC cells in meeting intracellular catabolic and anabolic demands. Mitochondrial stress testing revealed that NR4A1 knockout caused a defect in mitochondrial respiration, accompanied by intracellular oxidative homeostasis dysfunction. Notably, the redox equilibrium is sensitive to changes in any of the elements. Furthermore, BC cells lacking NR4A1 have greater lipid peroxidation than wild-type cells with endogenous NR4A1 expression. Lipid peroxidation is observed across cancer types and has been reported to be a poor prognostic factor for patients with BC^59^. Here, we detail the link between NR4A1 and lipid peroxidation in the progression of BC.

As a type of immediate-early protein, c-Fos dimerizes with nuclear proteins of the c-Jun family to interact with a specific *cis*-acting DNA sequence, the AP-1 motif, to regulate target gene transcription^60^. The transcriptional targets and functions are highly context dependent and depend on the partner with which c-Fos interacts. To identify the precise downstream effectors of NR4A1 and c-Fos, RNA-seq and ChIP–seq were performed in our study. Genome-wide analysis revealed the competitive occupancy of NR4A1 and c-Fos on the regulatory region of the critical target gene PRDX6. NR4A1 deficiency altered the gene network related to lipid metabolism by increasing the transcriptional activity of c-Fos to upregulate the expression of PRDX6, which led to the uncontrolled growth of BC. PRDX6 is overexpressed in multiple human cancers^61^. Notably, as a bifunctional protein, PRDX6 plays a critical role in tumor growth^62,63^. In our study, lipid metabolism remodeling and redox balance disruption in NR4A1-knockout BC cells may have resulted from the overexpression of PRDX6, which influences phospholipid metabolism as well as oxidative stress regulation.

In recent years, there has been a growing demand to investigate and develop targeted therapeutic drugs as strategies to treat multiple cancers in addition to chemotherapy and radiotherapy. The NR4A1-targeting agonist Csn-B, which was isolated from an endophytic fungus^64^, has been reported to have antitumor activity in colorectal cancer, gastric cancer and lung cancer^65–68^. In this study, we verified that Csn-B could suppress BC cell growth by upregulating the expression of NR4A1 and increasing the interaction abundance between NR4A1 and c-Fos to impede the transcriptional activity of c-Fos on PRDX6 through disruption of the association with chromatin. Interestingly, the antitumor effect of Csn-B was also reported in BC based on the alleviation of the NR4A1–PPARγ interplay and PPARγ-mediated NR4A1 degradation to augment NR4A1 function^25^. These results suggest that Csn-B may be a potent and effective agent for the treatment of BC. Furthermore, other compounds that target NR4A1, such as bisindole methane compounds and celastrol, have been reported to inhibit the development of various tumors and diseases^16,69^; these findings inspire us to further explore the detailed regulatory mechanisms involved in the treatment of BC in an NR4A1-dependent manner.

Although the expression of NR4A1 is downregulated in BC tissues, the underlying mechanism is unclear. In addition to the use of agonists, increasing the expression of NR4A1 in cells might be a more efficient strategy for exerting the tumor-suppressive effect of NR4A1 in BC. Epigenetic control-mediated transcriptional reprogramming is one of the major mechanisms that directly regulates gene expression, especially the crosstalk between transcription factors and DNA methylation, to induce the dysregulated transcription of target genes. Here, we uncover the potential mechanism responsible for the reduced NR4A1 expression in BC in which c-Fos cooperates with DNA methylation to generate a hypermethylated CpG island to inactivate the transcription of NR4A1. In addition, we confirmed the pharmacological effects of a DNA methylation inhibitor in restoring the expression of NR4A1 in BC cells. Due to the lack of specificity of such DNA methylation inhibitors in inhibiting the DNA methylation status at the genome-wide scale, the clinical application of these nonspecific DNA methylation inhibitors might be limited. Hence, interfering with the interaction between c-Fos and DNA hypermethylation might be a promising strategy to increase the efficiency of NR4A1 expression. However, c-Fos cannot directly catalyze DNA methylation. In fact, DNA methylation is mediated by DNA methyltransferases (DNMTs), including DNMT1, DNMT3A and DNMT3B, and the interaction between DNMTs and transcription factors is crucial in catalyzing targeted DNA methylation at specific genomic loci^70^. During this process, the transcription factor can recognize the targeted genomic site and recruit DNMTs to establish DNA methylation. c-Fos can interact with DNMT3A and DNMT3B to modulate DNMT3A/B-catalyzed targeted DNA hypermethylation in mammalian cells^70^. Our present study revealed that c-Fos access to the NR4A1 promoter is required for DNA hypermethylation of the NR4A1 promoter and the silencing of NR4A1 transcription. Thus, we propose that c-Fos might interact with DNMT3A and/or DNMT3B to recruit DNMT3A/B to catalyze the hypermethylation of the NR4A1 promoter, suggesting that impeding the interaction between c-Fos and DNMT3A/B might be an efficient approach to restore NR4A1 expression in the context of DNA hypermethylation, which needs to be further explored.

Taken together, our transcriptomic, metabolomic and epigenomic analyses provided details of the fundamental transcriptional function of NR4A1 in BC progression at the genome‐wide level. These observations highlight the complexity of the biological interplay among transcription factors in BC cells. Our findings may have important implications in finding therapeutic strategies to ameliorate BC progression.

## Supplementary information

Supplementary Information

## Data Availability

The data that support the findings of this study are included in the Article or its Supplementary Information. The unprocessed gel bots are provided in Supplementary Fig. 10. The high-throughput sequencing data have been deposited in the Gene Expression Omnibus database (https://www.ncbi.nlm.nih.gov/geo) with the accession number GSE281571 for RNA-seq and GSE281573 for ChIP–seq.
