# Supplementary material for: NR4A1 suppresses breast cancer growth by repressing c-Fos-mediated lipid and redox dyshomeostasis
Source: Exp Mol Med. 2025 Apr 1;57(4):804–19. doi: 10.1038/s12276-025-01430-3 (PMC12045962; doi:10.1038/s12276-025-01430-3)
Supplement: Supplementary file 1 — Supplementary Information [file 12276_2025_1430_MOESM1_ESM.pdf]

Supplementary Information for

NR4A1 suppresses breast cancer growth by repressing c-Fos-mediated lipid and redox dyshomeostasis

Cen Jiang✉, Youzhi Zhu, Junsi Zhang, Huaying Chen, Weiwei Li, Ruiwang Xie, Lingjun Kong, Ling Chen, Xiangjin Chen, Huifang Huang✉, Sunwang Xu✉

Contents:

Supplementary Fig. 1 DNA hypermethylation represses NR4A1 expression in BC, related to Figure 1.

Supplementary Fig. 2 NR4A1 overexpression inhibits the proliferation of BC cells, related to Figure 2.

Supplementary Fig. 3 NR4A1 silencing generates the aggressive phenotype of normal mammary epithelial cells, related to Figure 2.

Supplementary Fig. 4 NR4A1 silencing generates the aggressive phenotype of normal mammary epithelial cells, related to Figure 3.

Supplementary Fig. 5 NR4A1 silencing activates the expression of lipid metabolic regulated genes, related to Figure 5.

Supplementary Fig. 6 NR4A1 competitively inhibits c-Fos binding to targeted genes in BC cells, related to Figure 6.

Supplementary Fig. 7 NR4A1 inhibiting c-Fos-mediated transcription activation is independent on NR4A1 binding to target genes, related to Figure 6.

Supplementary Fig. 8 NR4A1 agonist Csn-B activate NR4A1 expression, related to Figure 7.

Supplementary Fig. 9 c-Fos inhibits NR4A1 transcription, related to Figure 8.

Supplementary Fig. 10 Unprocessed gel blot.

Supplementary Table 1 Altered metabolites in NR4A1-knockout MCF7 cells.

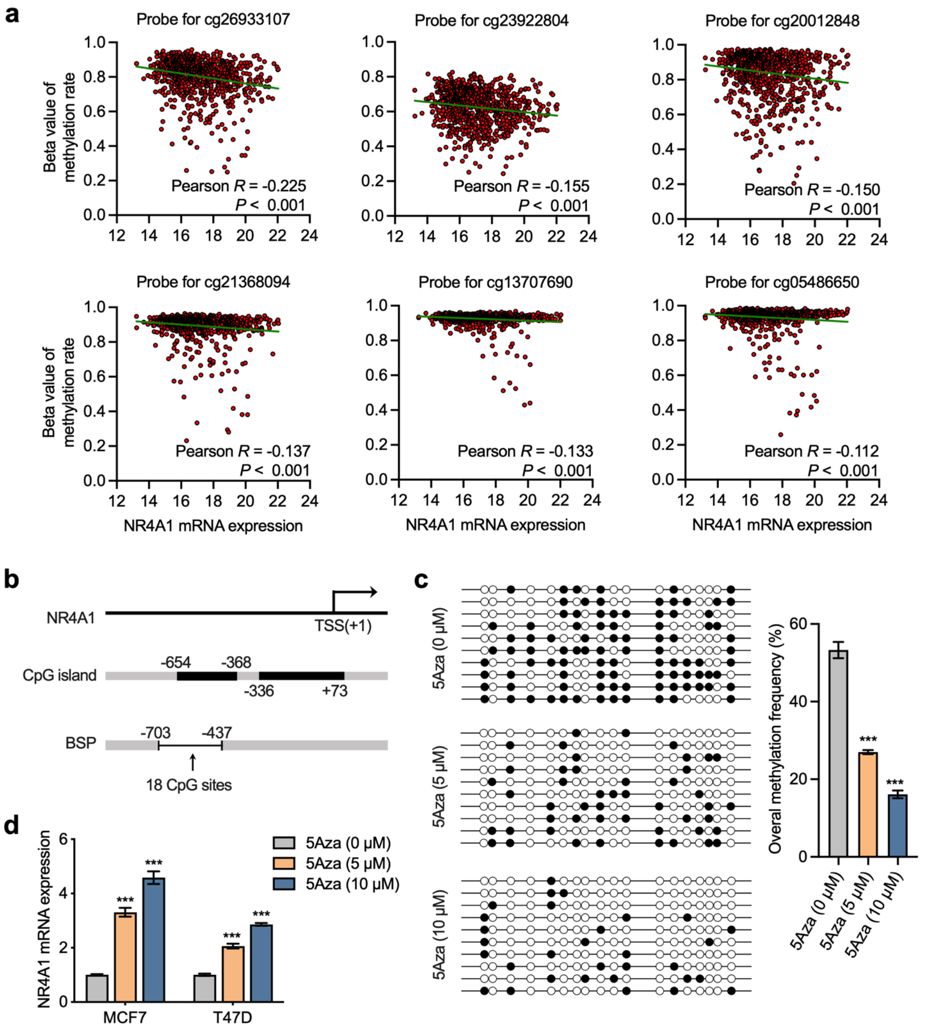

Supplementary Fig. 1 DNA hypermethylation represses NR4A1 expression in BC, related to Figure 1. a Correlation between the methylation level of multiple probes within NR4A1 genome and the transcription level of NR4A1 in breast cancer tissues in TCGA database. The linear correlation was determined by Pearson Correlation Coefficient. **b** Schematic diagram of CpG island on NR4A1 gene promoter region around the transcription start site (TSS). **c** Bisulfite sequencing PCR (BSP) assay was performed to analyze the methylation status of NR4A1 promoter in MCF7 cells treated with different concentrations of 5Aaz (5-Azacytidine, DNA methylation inhibitor). 18 individual CpG sites within the CGI were sequenced. Open circle indicates unmethylated CpG site and filled circle indicates methylated CpG site. The bar graph depicted the overall methylation rate of NR4A1 promoter. **d** RT-qPCR analysis of NR4A1 transcriptional levels in MCF7 and T47D cells treated with different concentrations of 5Aaz. Unpaired Student's *t*-tests were used in c, d, ***$P <$ 0.001.

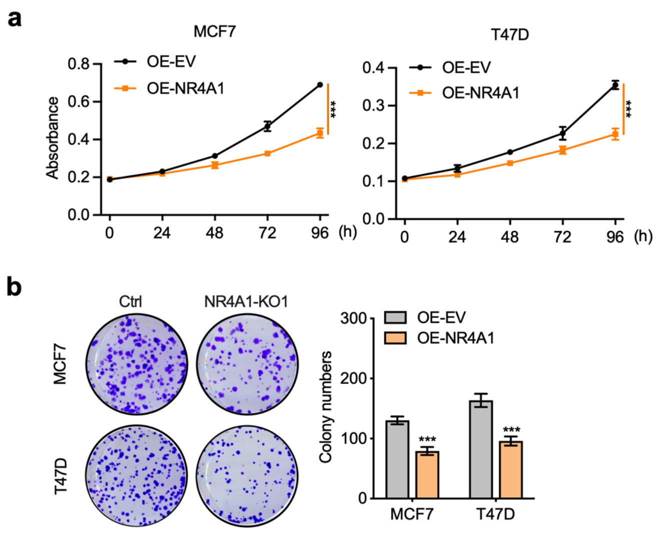

Supplementary Fig. 2 NR4A1 overexpression inhibits the proliferation of BC cells, related to Figure 2. a Cell proliferation assay was performed by CCK-8 assay in NR4A1-overexpressed and vector control MCF7 cells or T47D cells. One-way ANOVA, ****P* < 0.001. **b** Colony formation assay was performed in NR4A1-overexpressed and vector control MCF7 cells or T47D cells. Unpaired Student's *t*-tests, ****P* < 0.001.

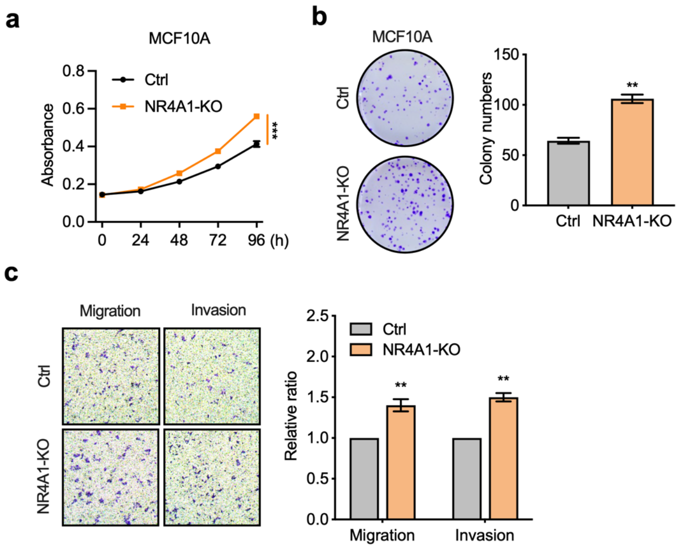

Supplementary Fig. 3 NR4A1 silencing generates the aggressive phenotype of normal mammary epithelial cells, related to Figure 2. a Cell proliferation assay was performed by CCK-8 assay in NR4A1-knockout and parental control MCF10A cells. One-way ANOVA, ***$P < 0.001$ **b** Colony formation assay was performed in NR4A1-knockout and parental control MCF10A cells. Unpaired Student's *t*-tests, ***$P < 0.001$. **c** Representative images (left) and statistical bar graphs (right) depicting the relative cell migration and invasion rate in MCF10A cells with or without NR4A1 knockout. Paired Student's *t*-tests, **$P < 0.01$.

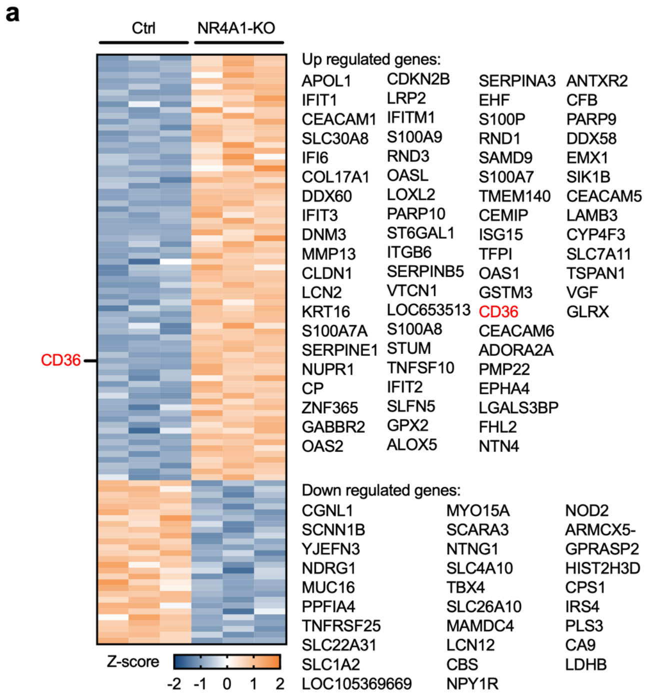

Supplementary Fig. 4 NR4A1 silencing generates the aggressive phenotype of normal mammary epithelial cells, related to Figure 3. a Heatmap showed the 73 increased and 28 decreased genes (fold change > 1.5, FDR < 0.05) in NR4A1-knockout MCF7 cells compared to parental MCF7 cells.

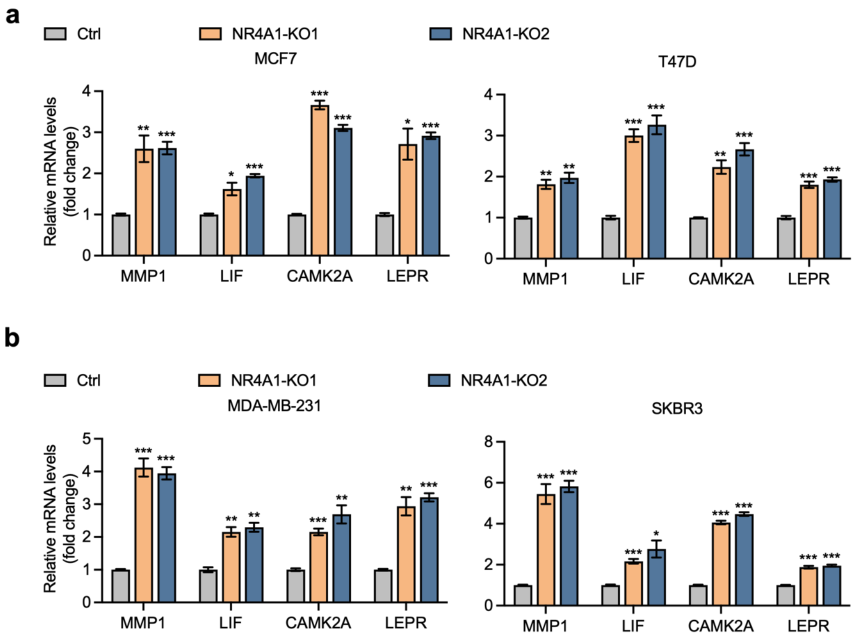

Supplementary Fig. 5 NR4A1 silencing activates the expression of lipid metabolic regulated genes, related to Figure 5. a RT-qPCR analysis of the transcriptional levels of lipid metabolic regulated genes in NR4A1-knockout and parental MCF7 or T47D cells, which represent for luminal BC subtypes. **b** RT-qPCR analysis of the transcriptional levels of lipid metabolic regulated genes in NR4A1-knockout and parental MDA-MB-231 or SKBR3 cells, which represent for basal BC subtypes and HER2 positive BC subtypes, respectively. Unpaired Student's *t*-tests were used in **a**, **b**, **P* < 0.05, ***P* < 0.01, ****P* < 0.001.

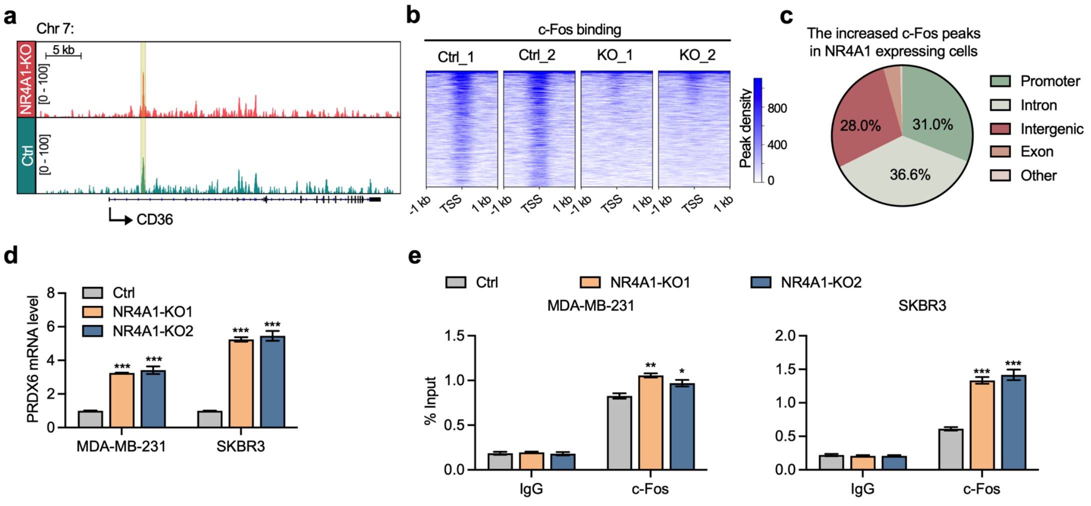

Supplementary Fig. 6 NR4A1 competitively inhibits c-Fos binding to targeted genes in BC cells, related to Figure 6. a c-Fos ChIP-seq tracks at CD36 gene locus. **b** Heat maps of c-Fos ChIP-seq signals sorted on the basis of increased c-Fos peaks between parental control and NR4A1-knockout MCF7 cells. **c** Genomic annotations of the increased c-Fos peaks in MCF7 cells with NR4A1 expressing by chromosome location. **d** RT-qPCR analysis of PRDX6 mRNA levels in NR4A1 knockout and parental control basal like MDA-MB-231 and HER2 positive SKBR3 cells. **e** ChIP-qPCR analysis of c-Fos enrichment on PRDX6 promoter in NR4A1 knockout and parental control basal like MDA-MB-231 and HER2 positive SKBR3 cells. Unpaired Student's *t*-tests were used in **d**, **e**, *$P < 0.05$, **$P < 0.01$, ***$P < 0.001$.

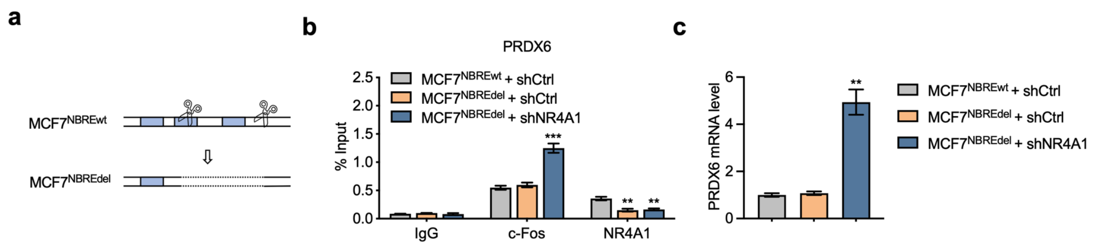

Supplementary Fig. 7 NR4A1 inhibiting c-Fos-mediated transcription activation is independent on NR4A1 binding to target genes, related to Figure 6. a Schematic diagram showing the genome-editing strategy to delete the NBRE-like elements on PRDX6 promoter in MCF7 cells. **b** ChIP assays of the c-Fos and NR4A1 enrichment on PRDX6 promoter in MCF7 cells with or without NBRE-like elements deleted on PRDX6 promoter and transfected with NR4A1 targeted shRNA or no targeted control shRNA. **c** RT-qPCR analysis of PRDX6 mRNA levels in MCF7 cells with or without NBRE-like elements deleted on PRDX6 promoter and transfected with NR4A1 targeted shRNA or no targeted control shRNA. Unpaired Student's *t*-tests were used in **b**, **c**, $^{**}P < 0.01$, $^{***}P < 0.001$.

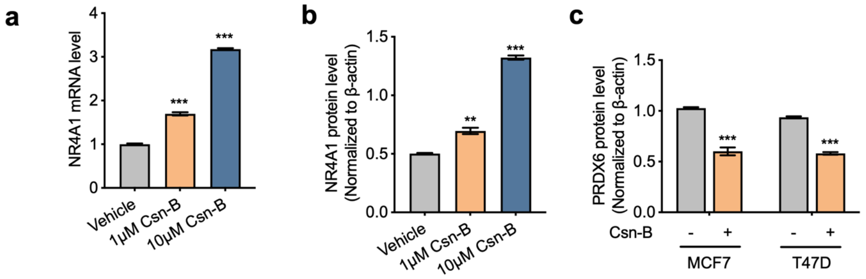

Supplementary Fig. 8 NR4A1 agonist Csn-B activate NR4A1 expression, related to Figure 7. **a** RT-qPCR analysis of NR4A1 transcriptional levels in MCF7 cells treated with different concentrations of Csn-B. **b** Statistical analysis of NR4A1 protein levels in MCF7 cells treated with different concentrations of Csn-B (related to Fig. 7a), measured with grayscale value by Image J and normalized to β-actin. **c** Statistical analysis of NR4A1 protein levels in MCF7 and T47D cells treated with or without Csn-B (related to Fig. 7k), measured with grayscale value by Image J and normalized to β-actin. Unpaired Student's *t*-tests were used in **a**, **b**, **c**, $^{**}P < 0.01$, $^{***}P < 0.001$.

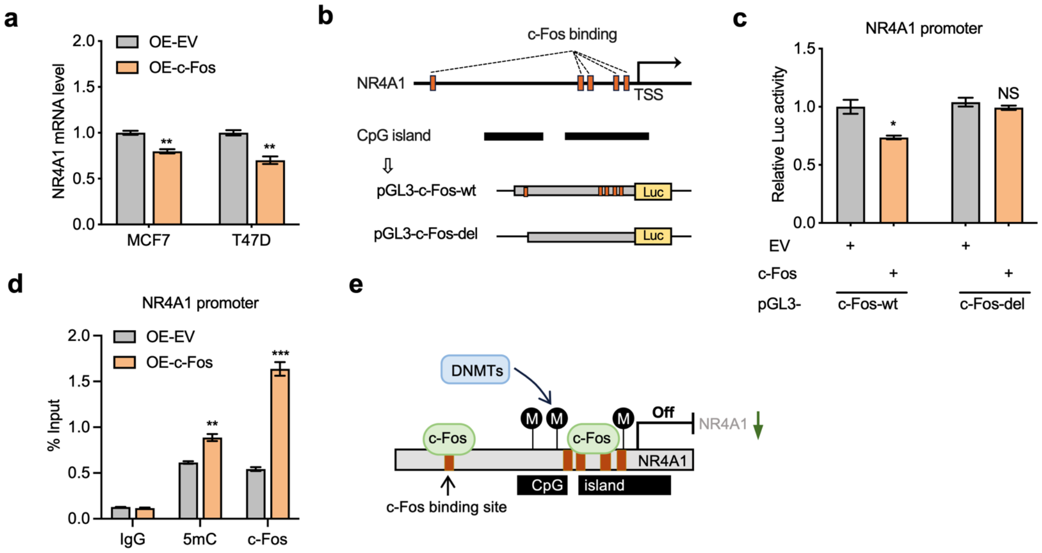

Supplementary Fig. 9 c-Fos inhibits NR4A1 transcription, related to Figure 8. a RT-qPCR analysis of NR4A1 transcriptional levels in MCF7 and T47D cells with c-Fos overexpression of vector control. **b** Schematic diagram of NR4A1 promoter showing c-Fos binding motifs and CpG islands in the regulator region. pGL3-c-Fos-wt and pGL3-c-Fos-del stand for the NR4A1 promoter region with c-Fos binding sites or c-Fos binding sites-deleted sequences, which were cloned upstream of the firefly luciferase gene in the pGL3-basic vector. **c** NR4A1 promoter constructs were co-transfected with c-Fos or empty vector (EV) to detect luciferase activity in HEK293T cells. pRL-TK was transfected for normalization, and luciferase activity was measured by using a dual luciferase reporter assay system. **d** ChIP-qPCR analysis of c-Fos enrichment and DNA methylation signal 5mC occupancy on NR4A1 promoter in c-Fos-overexpressed and vector control MCF7 cells. **e** Schematic diagram for c-Fos cooperates with DNA methylation to inhibit NR4A1 expression in BC. Unpaired Student's *t*-tests were used in **a**, **c**, **d**, *$P < 0.05$, **$P < 0.01$, ***$P < 0.001$, NS non-significant.

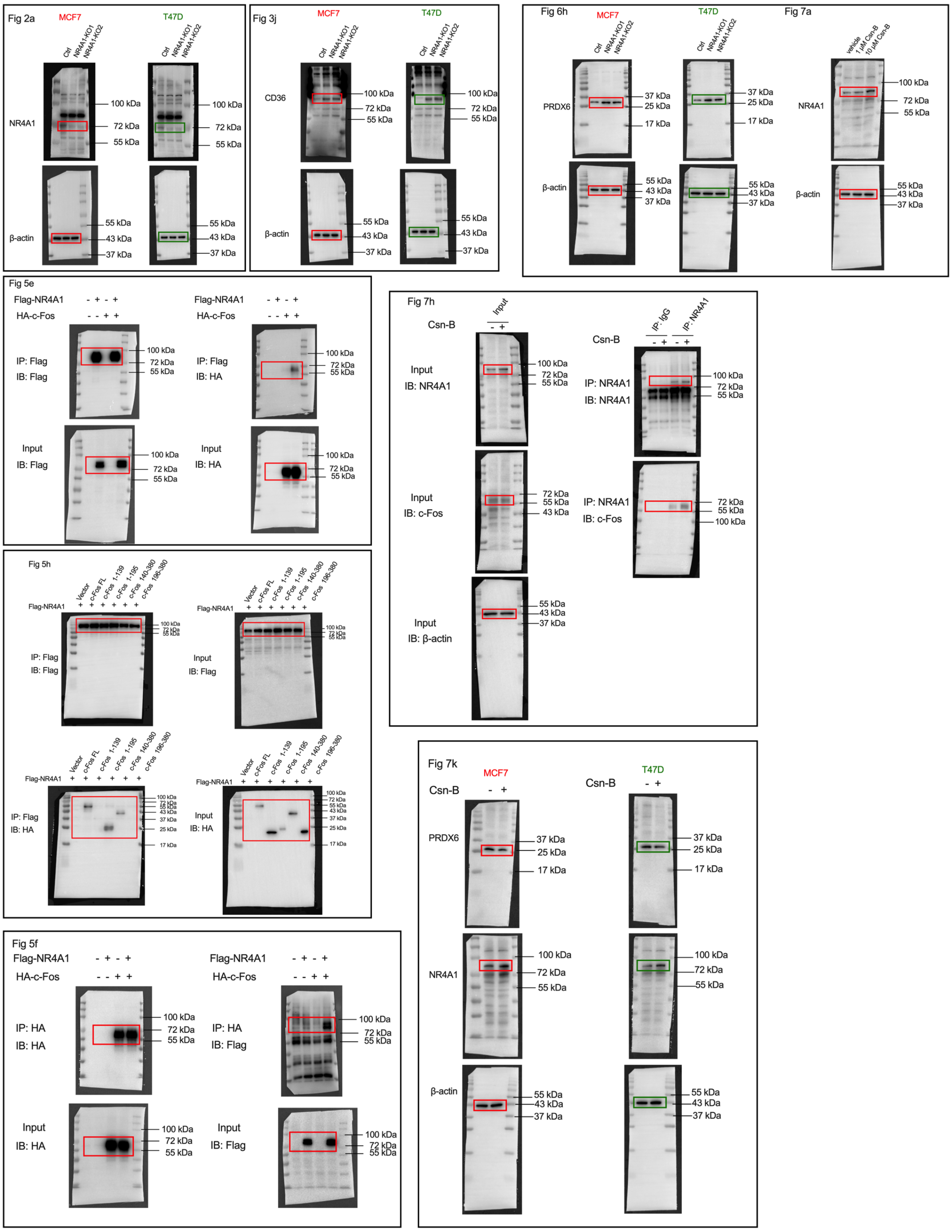

Supplementary Fig. 10 Unprocessed gel blot.

Supplementary Table 1 Altered metabolites in NR4A1-knockout MCF7 cells

Index	Formula	Compounds	log2(FC)	FDR
ZINC388251	C8H10O2	1,2-Dimethoxybenzene	-1.3852	0.020506
MW0169964	C20H24O3	TRIPTOPHENOLIDE	-1.7577	0.010345
MW0169549	C6H6N2O	Niacinamide	1.1063	0.023547
MW0169241	C10H13N5O5	GUANOSINE	-1.9826	0.023813
MW0168250	K2HPO4	Potassium dibasic phosphate	-0.70732	0.0015775
MW0164178	C23H48NO7P	2-azaniumylethyl [(2R)-2-hydroxy-3-octadecanoyloxypropyl] phosphate	-1.0812	0.0079126
MW0158467	C25H39N5O8	Tyr-Glu-Val-Lys	-0.69981	0.0021089
MW0157826	C20H28N4O5S1	Thr-Trp-Met	-0.89501	0.00063826
MW0156741	C23H35N7O8	Ser-Glu-Phe-Arg	-1.1107	0.002416
MW0155355	C25H42N6O6	Phe-Thr-Lys-Lys	-0.69345	0.004704
MW0155158	C24H36N8O8	Phe-Gln-Asp-Arg	-0.96447	0.014779
MEDN0074	C8H14N2O5S	gamma-L-Glutamate-Cysteine	0.66352	0.0051066
MW0153022	C23H46N6O5	Lys-Ile-Val-Lys	-1.034	0.0010266
MW0152703	C28H48N4O3S	Linoleic Acid-biotin	-2.2459	0.006466
MW0152272	C27H46N8O8	Leu-Asp-Val-Lys-His	-0.75674	0.0040515
MW0151990	C21H40N2O2S	Kalkitoxin thioamide alcohol	-0.90908	0.002635
MW0150853	C16H26N6O5	His-Asn-Ile	-0.97526	0.0017162
MW0148532	C16H25N5O6	Dihydrozeatin-O-glucoside	-1.7516	0.0065025
MW0148485	C35H44N2O9	Dihydro Isorescinnamine	-2.0354	0.00068508
MW0108634	C6H9NO5	N-Acetyl-L-aspartic acid	0.79909	0.00022446
MW0147117	C25H45N3O7S	Carmamycin B	-0.61196	0.00044254
MW0145679	C25H41N9O6	Asn-Arg-Phe-Lys	-0.94851	0.0023929
MW0145522	C22H45N11O6	Arg-Thr-Lys-Arg	-1.065	0.0061911
MW0145452	C16H32N6O4S2	Arg-Met-Met	-0.67796	0.010832
MW0145409	C22H43N11O6	Arg-Leu-Asn-Arg	-1.3337	0.00061451
MW0145360	C24H44N8O8	Arg-Gly-Glu-Val-Leu	-0.75474	0.010149
MW0145298	C28H44N8O10	Arg-Asp-Leu-Tyr-Ser	-0.64536	0.018751
MW0144288	C36H45NO13	Aclacinomycin S	-0.58768	0.0035602
MW0108608	C11H16N2O8	N-Acetyl-Asp-Glu	1.6784	0.0020258
MW0105103	C7H11N3O2	3-Methylhistidine	0.58659	0.00043079
MW0107943	C5H9NO2	L-Proline	0.66531	0.00012014
MW0120491	C16H14N2O2	4H-3,1-Benzoxazin-4-one,6-methyl-2-[(4-methylphenyl)amino]-	0.64393	0.00010946
MW0138365	C39H44N2O7	Hernandezine	-0.62963	0.013037
MW0009758	C12H20N2O3S	Sotalol	0.65647	0.00025229
MW0130875	C10H10O2	2-Methoxycinnamaldehyde	-1.1384	0.015994
MW0130336	C18H20O4	2,3',4,5'-Tetramethoxystilbene	-1.0747	0.0023929
MW0126557	C25H38O5	Simvastatin	-1.1042	0.0018503
MEDP1218	C11H9NO2	3-Aminonaphthalene-2-carboxylic acid	0.58803	0.0040515

MW0126295	C5H4N2O2	Pyrazine-2-carboxylic acid	-0.65213	0.0075796
MEDN1654	C15H22O3	Gemfibrozil	1.3492	0.0027331
MW0126012	C5H4N4O2	Oxypurinol	-0.67823	0.0065025
MW0125153	C7H7NO2	Methyl Nicotinate	1.0505	2.96E-06
MW0154314	C47H74N4O8	NBD-Stearoyl-2-Arachidonoyl-sn-glycerol	1.0247	0.028679
MW0122903	C30H42N2O2	Arachidonoyl Serotonin	-0.61429	0.0017419
MW0012996	C43H72O5	1-Stearoyl-2-docosahexaenoyl-sn-glycerol	0.60908	0.031626
MW0120634	C4H5NS	4-Methylthiazole	-1.3684	0.0093516
MW0138912	C16H14O4	Medicarpin	0.59215	6.25E-06
MW0119782	C6H8S2	3-Vinyl-1,2-dithiacyclohex-4-ene	-1.0033	0.030814
MW0118552	C30H44N6O2	2H-1-Benzopyran-6-ol,2-((4-(2,6-di-1-pyrrolidinyl-4-pyrimidinyl)-1-piperazinyl)methyl)-3,4-dihydro-2,5,7,8-tetramethyl-,dihydrochloride,(2R)-	-1.0604	0.0025359
MW0117273	C21H22N4O	1-Piperazinecarboxamide,N-phenyl-4-(2-quinolinylmethyl)-	-1.2586	0.0017419
MW0114584	C8H19NO	Heptaminol	-1.3265	0.0021593
MW0111849	C19H37O7P	1-Palmitoylglycerone 3-phosphate	-1.3802	0.0036265
MW0137870	C20H14O7	Daphnoretin methyl ether	0.6094	0.00025229
MW0111235	C18H39N	Octadecylamine	-1.2823	0.015994
MW0126448	C17H20N4O6	Riboflavin	0.74162	2.32E-06
MW0110983	C20H41NO2	D-erythro-Sphingosine C-20	-2.0483	0.0066261
MW0110020	C2HCl3O2	Trichloroacetic acid	-1.2518	0.0020669
MW0126097	C19H14O5S	Phenol red	0.8182	3.97E-05
MW0124374	C14H13ClN6O5S	Imazosulfuron	0.72886	0.00043079
MW0139217	C14H14O3	Osthenol	0.61495	0.00028858
MW0122713	C24H34N2O2	9-Octadecyn-1-one,1-oxazolo[4,5-b]pyridin-2-yl-	5.3245	0.0039097
MW0109524	C23H32N2O5	Ramipril	0.8894	3.97E-05
MW0109053	C16H18N2O4S	Penicillin G	0.66809	0.0003502
MW0105302	C5H11NO2	5-Aminovaleric Acid	-0.78199	0.00040185
MW0142110	C5H4N4O2	Xanthine	0.96385	0.0039734
MEDN0294	C7H11NO3	3-Methylcrotonyl Glycine	0.66906	0.00030326
MW0063590	C27H43NO2	Solasodine	-1.7629	0.010345
MW0063530	C27H43NO3	Sipeimine	-0.68181	0.00025229
MW0063477	C20H34O2	Sciadonic acid	-0.72248	0.0023915
MW0062164	C21H32O2	Pregnenolone	-0.88044	0.0078292
MEDN0284	C6H12O3	2-Hydroxy-4-methylvaleric acid	1.107	0.00043079
MW0056221	C44H81O8P	PA(22:1(13Z)/19:2(10Z,13Z))	2.0384	0.030034
MW0060586	C49H84NO8P	PE-NMe2(20:2(11Z,14Z)/22:5(4Z,7Z,10Z,13Z,16Z))	-2.6848	0.016134
MW0056213	C41H77O8P	PA(22:1(13Z)/16:1(9Z))	2.0172	0.014794
MW0055917	C42H83O8P	PA(20:0/i-19:0)	2.038	0.032542
MW0055915	C40H79O8P	PA(20:0/i-17:0)	1.4529	0.010605
MW0055907	C40H79O8P	PA(20:0/a-17:0)	4.6779	0.00040702
MW0055877	C40H79O8P	PA(20:0/17:0)	2.5095	0.0066342

MW0055735	C43H81O8P	PA(18:1(9Z)/22:1(13Z))	1.5935	0.045464
MW0055697	C41H79O8P	PA(18:1(11Z)/20:0)	3.2496	0.0064378
MW0055671	C41H75O8P	PA(18:0/20:3(5Z,8Z,11Z))	1.9774	0.0077482
MW0055558	C39H75O8P	PA(14:0/22:1(13Z))	1.124	0.029171
MW0148480	C44H84NO7P	Dihomo--Linolenoyl PAF C-16	3.3697	0.0069973
MW0141122	C44H84NO8P	1,2-Dioleoyl PC	3.8129	0.021847
MW0057352	C46H80NO8P	PC(20:4(5Z,8Z,11Z,14Z)/18:2(9Z,12Z))	1.9953	0.035209
MW0057248	C44H84NO8P	PC(20:2(11Z,14Z)/16:0)	2.9005	0.0015817
MW0057215	C44H86NO8P	PC(20:1(11Z)/16:0)	1.6629	0.00025229
MW0057180	C42H82NO8P	PC(20:0/14:1(9Z))	4.265	0.00063826
MW0057085	C44H82NO8P	PC(18:3(6Z,9Z,12Z)/18:0)	1.3305	0.0019213
MW0059313	C43H84NO8P	PE-NMe(15:0/22:1(13Z))	-2.895	0.016279
MW0057062	C46H82NO8P	PC(18:2(9Z,12Z)/20:3(5Z,8Z,11Z))	0.85446	0.041221
MW0057020	C44H84NO8P	PC(18:1(9Z)/18:1(9Z))	1.0447	0.04272
MW0057015	C41H80NO8P	PC(18:1(9Z)/15:0)	2.6702	0.020915
MW0056986	C44H84NO8P	PC(18:1(11Z)/18:1(11Z))	1.0449	0.022433
MW0056985	C44H86NO8P	PC(18:1(11Z)/18:0)	3.1199	0.017319
MW0056927	C44H84NO8P	PC(16:1(9Z)/20:1(11Z))	2.431	0.0087449
MW0056895	C44H82NO8P	PC(16:0/20:3(5Z,8Z,11Z))	1.9795	0.044272
MW0056892	C44H88NO8P	PC(16:0/20:0)	2.5084	0.019966
MW0058084	C36H72NO8P	PE(15:0/16:0)	-3.0599	0.038845
MW0056852	C41H82NO8P	PC(15:0/18:0)	6.0847	0.0119
MW0056834	C44H86NO8P	PC(14:1(9Z)/22:0)	1.1121	0.01299
MW0056798	C42H76NO8P	PC(14:0/20:4(5Z,8Z,11Z,14Z))	4.085	0.00078857
MW0052514	C44H80NO7P	Eicosapentaenoyl PAF C-16	2.4955	0.020268
MW0011883	C44H80NO8P	1,2-Dilinoleoyl-sn-glycero-3-phosphocholine	4.0661	0.022312
MW0011790	C48H84NO7P	1-(1Z-Octadecenyl)-2-(4Z,7Z,10Z,13Z,16Z,19Z-docosahexaenoyl)-sn-glycero-3-phosphocholine	1.3289	0.025038
MW0060673	C49H80NO8P	PE-NMe2(20:4(5Z,8Z,11Z,14Z)/22:5(4Z,7Z,10Z,13Z,16Z))	5.6653	0.0012233
MW0060587	C49H84NO8P	PE-NMe2(20:2(11Z,14Z)/22:5(7Z,10Z,13Z,16Z,19Z))	4.3052	0.0010266
MW0060407	C45H76NO8P	PE-NMe2(18:2(9Z,12Z)/20:5(5Z,8Z,11Z,14Z,17Z))	1.7525	0.0040515
MW0060367	C43H80NO8P	PE-NMe2(18:1(9Z)/18:2(9Z,12Z))	1.5065	0.011039
MW0060278	C41H78NO8P	PE-NMe2(16:1(9Z)/18:1(11Z))	1.297	0.020411
MW0056905	C46H82NO8P	PC(16:0/22:5(7Z,10Z,13Z,16Z,19Z))	-1.0367	0.039553
MW0060227	C42H82NO8P	PE-NMe2(15:0/20:1(11Z))	2.077	0.046851
MW0060220	C40H78NO8P	PE-NMe2(15:0/18:1(11Z))	2.8515	0.0016246
MW0056883	C40H80NO8P	PC(16:0/16:0)	-1.3201	0.03908
MW0060197	C41H80NO8P	PE-NMe2(14:1(9Z)/20:0)	2.4855	0.0014996
MW0060169	C41H80NO8P	PE-NMe2(14:0/20:1(11Z))	1.8498	0.0012233
MW0059613	C41H80NO8P	PE-NMe(20:1(11Z)/15:0)	2.1421	0.04086
MW0059584	C41H82NO8P	PE-NMe(20:0/15:0)	1.8598	0.0017162
MW0059450	C44H84NO8P	PE-NMe(18:1(9Z)/20:1(11Z))	1.9669	0.0075803

MW0059423	C44H80NO8P	PE-NMe(18:1(11Z)/20:3(5Z,8Z,11Z))	2.3186	0.0022003
MW0059386	C42H82NO8P	PE-NMe(18:0/18:1(9Z))	1.1687	0.023538
MW0059385	C42H82NO8P	PE-NMe(18:0/18:1(11Z))	4.0474	0.00017348
MW0059344	C44H80NO8P	PE-NMe(16:0/22:4(7Z,10Z,13Z,16Z))	2.3845	0.0014939
MW0059343	C44H84NO8P	PE-NMe(16:0/22:2(13Z,16Z))	2.8128	0.00016884
MW0055698	C41H77O8P	PA(18:1(11Z)/20:1(11Z))	-0.59216	0.00077643
MW0059314	C43H82NO8P	PE-NMe(15:0/22:2(13Z,16Z))	5.6298	0.019181
MW0059306	C41H78NO8P	PE-NMe(15:0/20:2(11Z,14Z))	3.4934	0.0046938
MW0059304	C41H82NO8P	PE-NMe(15:0/20:0)	6.3078	0.00043079
MW0054508	C27H53O7P	LysoPA(24:1(15Z)/0:0)	-1.38	0.0019483
MW0054485	C19H39O7P	LysoPA(16:0/0:0)	-0.82251	0.00021505
MW0054217	C32H58NO11P	KOdiA-PC	-0.75749	0.0054669
MW0053722	C21H38O6	Glycerol trihexanoate	-1.3207	0.022355
MW0052908	C23H36N2O2	Finasteride	-0.85826	0.0016562
MW0052880	C17H28O2	Farnesyl acetate	-0.71049	0.00064279
MW0059283	C42H82NO8P	PE-NMe(14:1(9Z)/22:0)	1.5954	0.03316
MW0052486	C35H66O8	Donhexocin	-0.68197	0.00043079
MW0050743	C47H82O5	DG(20:5(5Z,8Z,11Z,14Z,17Z)/24:0/0:0)	-2.8616	0.004294
MW0050174	C39H66O5	DG(18:2(9Z,12Z)/18:3(9Z,12Z,15Z)/0:0)	-3.3918	0.0065025
MW0049861	C37H62O5	DG(16:1(9Z)/18:4(6Z,9Z,12Z,15Z)/0:0)	-0.62475	0.0007867
MW0017203	C33H44O	Citranaxanthin	-1.891	0.011827
MW0017116	C26H42O11	Cinncassiol D2 glucoside	-0.63655	0.0088385
MW0016182	C24H28O2	Bexarotene	-1.4016	0.0052484
MW0015862	C44H82NO7P	Arachidonoyl PAF C-16	-0.81118	0.0044835
MW0015651	C32H44O2	all-trans-Carophyll yellow	-0.8928	0.0024656
MW0058713	C42H82NO8P	PE(22:1(13Z)/15:0)	1.03	0.0098832
MW0015586	C22H36O2	Adrenic acid	-0.76079	0.00062551
MW0015283	C20H32O4	8,15-Dihete	-1.1378	4.69E-05
MW0014037	C39H58N4O5	3-hexanoyl-NBD Cholesterol	-1.8252	0.0017162
MW0013015	C14H30O	1-Tetradecanol	-1.8293	0.0052796
MW0058712	C41H78NO8P	PE(22:1(13Z)/14:1(9Z))	4.7433	0.0076024
MW0012982	C23H48NO7P	1-Pentadecanoyl-sn-glycero-3-phosphocholine	-0.68498	0.0035602
MW0012963	C21H44NO7P	1-Palmitoyl-2-hydroxy-sn-glycero-3-phosphoethanolamine	-1.3561	0.0016246
MW0058711	C41H80NO8P	PE(22:1(13Z)/14:0)	3.399	0.0043415
MW0012936	C23H42O5	1-Oleoyl-2-acetyl-sn-glycerol	-1.7953	0.0035602
MW0012931	C26H54NO7P	1-O-Hexadecyl-2-O-acetyl-sn-glyceryl-3-phosphorylcholine	-1.0251	0.0014439
MW0012929	C26H55N2O7P	Methylcarbamyl PAF C-16	-0.80625	0.0019213
MW0012806	C25H52NO7P	1-Heptadecanoyl-sn-glycero-3-phosphocholine	-0.72987	0.0020972
MW0012774	C28H58NO7P	1-Arachidoyl-2-hydroxy-sn-glycero-3-phosphocholine	-0.93182	0.00072731
MW0012743	C18H28O2	19-Noretiocholanolone	-0.7394	0.0023915
MW0012646	C23H28O4	17-phenyl trinor Prostaglandin A2	-0.68159	0.0016848

MW0012368	C23H37NO5S	14,15-Leukotriene E4	-0.62211	0.0029097
MW0012353	C22H43NO	13Z-Docosenamide	-1.89	8.95E-05
MW0058418	C41H80NO8P	PE(20:0/16:1(9Z))	1.484	0.023547
MW0011898	C19H36O5	1,2-Dioctanoyl-sn-glycerol	-0.73924	0.00077253
MW0058254	C41H78NO8P	PE(18:1(9Z)/18:1(11Z))	2.083	0.036853
MW0011798	C16H24O	1-(2,6,6-Trimethyl-2-cyclohexen-1-yl)-1,6-heptadien-3-one	-1.0053	0.0017419
MW0011795	C26H54NO6P	1-(1Z-Octadecenyl)-sn-glycero-3-phosphocholine	-1.0176	0.0019293
MW0011907	C42H80NO8P	1,2-Dioleoyl-sn-glycero-3-phosphoethanolamine-N-methyl	1.4321	0.033449
MW0011495	C32H42O	(all-E)-6'-Apo-y-caroten-6'-al	-1.4155	0.00063826
MW0011096	C34H50O6	(2xi,3xi)-2,3-Dihydroxy-12,18-ursadien-28-oic acid diacetate	-1.2997	0.0038844
MW0010776	C27H44O7	(+)-Ecdysterone	-0.72514	0.0031248
MW0010009	C26H43NO3	Undecanamide,N-cyclopropyl-11-(2-hexyl-5-hydroxyphenoxy)-	-0.66864	0.010149
MW0012955	C42H75O10P	1-Palmitoyl-2-arachidonoyl-sn-glycero-3-phospho-(1'-sn-glycerol)	1.882	0.037805
MW0009611	C19H25ClN2OS	Pyridaben	-1.3975	0.042868
MW0007865	C15H17ClN4	Myclobutanil	-0.60669	0.00043079
MW0007841	C12H14O4	Monoisobutyl phthalate	-0.60452	0.0020741
MW0006844	C24H38O4	Diisooctyl phthalate	-2.4804	0.0016029
MW0006560	C7H5Cl2NS	Chlorthiamid	-0.72915	0.0087066
MW0006140	C7H6O	Benzaldehyde	-0.78116	0.022312
MW0005470	C10H6O4	5,8-Dihydroxy-1,4-naphthoquinone	-0.80823	0.046092
MW0015610	C23H28O11	Albiflorin	0.84753	0.00063826
MW0000246	C31H39N5O5	Ergocornine	-0.86516	0.00043079
MEDTP01664	C21H41NO4	Carnitine C14:0	-0.74656	0.0015453
MEDP1838	C10H26N4	Spermine	-0.85059	0.033652
MEDP1685	C18H39NO3	Phytosphingosine	-0.63563	4.34E-05
MW0000720	C15H17I2NO2	Ioxynil octanoate	0.69691	0.00013634
MEDP1448	C21H43O4	MG(18:0/0:0/0:0)	-0.6869	0.041149
MEDP1446	C18H33NO	Octadecadienamide	-1.5582	0.014794
MEDP1271	C16H22O4	Bis(2-ethylhexyl) phthalate	-0.65636	0.0019213
MW0111314	C40H84N2O5PS	Thioetheramide PC	2.0896	0.034157
MEDP1170	C18H34O	9-octadecenal	-2.0414	0.010633
MEDP0574	C18H35NO	Oleamide	-1.9609	0.0025559
MW0111098	C21H46NO4P	Miltefosine	0.77684	0.00010946
MW0109301	C8H14N2O3	Pro-Ala	0.66872	0.0020741
MW0103993	C13H21N3O8S	(R)-S-Lactoylglutathione	0.99395	0.0040515
MEDN0167	C5H4N4	Purine	-1.0552	0.0013916
MEDP1664	C17H32N6O6	Glu-Leu-Arg	1.0173	1.22E-05
MEDL02503	C23H45NO4	Carnitine C16:0	-0.64481	0.001557

MEDL00391	C21H44NO7P	LPE(16:0/0:0)	-0.72677	0.00018672
